# Aircraft-Based AirCore Sampling for Estimates of N_2_O and CH_4_ Emissions

**DOI:** 10.1021/acs.est.3c04932

**Published:** 2023-10-05

**Authors:** Xin Tong, Steven van Heuven, Bert Scheeren, Bert Kers, Ronald Hutjes, Huilin Chen

**Affiliations:** † Centre for Isotope Research (CIO), Energy and Sustainability Research Institute Groningen (ESRIG), University of Groningen, 9747 AG Groningen, The Netherlands; ‡ Joint International Research Laboratory of Atmospheric and Earth System Sciences, School of Atmospheric Sciences, Nanjing University, 210023 Nanjing, China; § Water Systems and Global Change, Department of Environmental Sciences, Wageningen University and Research, 6708 PB Wageningen, The Netherlands

**Keywords:** AirCore, airborne measurements, N_2_O emissions, CH_4_ emissions, urban areas

## Abstract

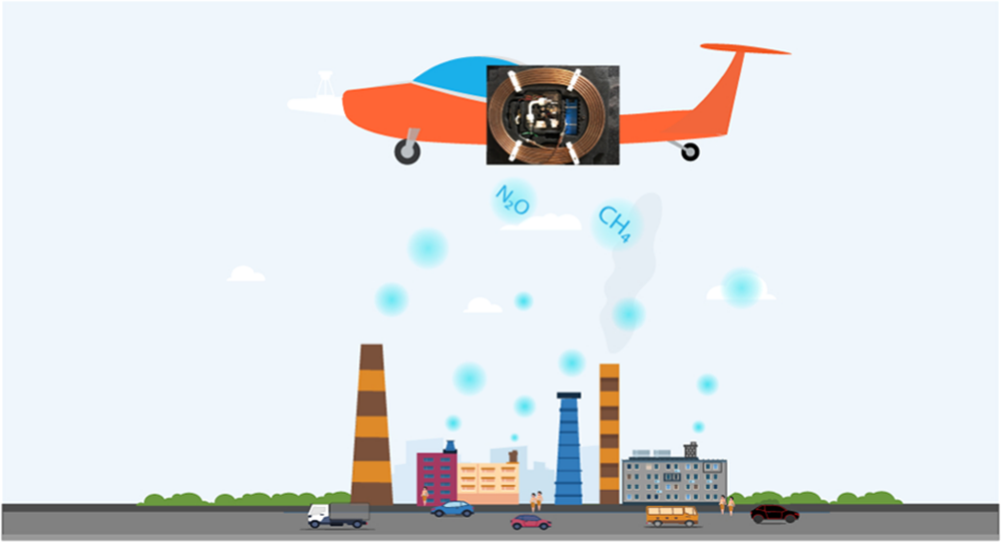

Airborne measurements offer an effective way to quantify urban emissions of greenhouse gases (GHGs). However, it may be challenging due to the requirement of high measurement precision and sufficiently enhanced signals. We developed a new active AirCore system based on the previous unmanned aerial vehicle (UAV) version, which is capable of sampling atmospheric air for several hours aboard a lightweight aircraft for postflight simultaneous and continuous measurements of N_2_O, CH_4_, CO_2_, and CO. We performed 13 flights over the urban areas of Groningen, Utrecht, and Rotterdam and evaluated the aircraft-based AirCore measurements against in situ continuous CH_4_ measurements. One flight was selected for each of the three urban areas to quantify the emissions of N_2_O and CH_4_. Compared to the Dutch inventory, the estimated N_2_O emissions (364 ± 143 kg h^–1^) from the Rotterdam area are ∼3 times larger, whereas those for Groningen (95 ± 90 kg h^–1^) and Utrecht (32 ± 16 kg h^–1^) are not significantly different. The estimated CH_4_ emissions for all three urban areas (Groningen: 2534 ± 1774 kg CH_4_ hr^–1^, Utrecht: 1440 ± 628 kg CH_4_ hr^–1^, and Rotterdam: 2419 ± 922 kg CH_4_ hr^–1^) are not significantly different from the Dutch inventory. The innovative aircraft-based active AirCore sampling system provides a robust means of high-precision and continuous measurements of multiple gas species, which is useful for quantifying GHG emissions from urban areas.

## Abstract 2

Innovative airborne AirCore sampling reveals significant underestimation of N_2_O emissions in Rotterdam, which provides a useful tool to evaluate urban greenhouse gas emissions.

## 1 Introduction

Nitrous oxide (N_2_O) and methane (CH_4_) have a global warming potential (GWP) of 273 and 30 times that of carbon dioxide (CO_2_) over a 100-year time frame, respectively.^1^ Current emissions of N_2_O lead to a long-term impact on climate because N_2_O can persist in the atmosphere for 109 years^1^ and is the primary ozone-depleting substance throughout the 21st century.^2^ CH_4_ has a relatively short lifetime of 11.8 years compared to N_2_O, and its GWP for CH_4_ increases to 83 over a 20-year time frame,^1^ which is considered by many to be a more relevant time frame, given the urgency of emission reductions. Accurately quantifying the emissions of N_2_O and CH_4_ is crucial for making effective mitigation policies, which is, however, challenging due to the high spatiotemporal variability of their fluxes. Emission estimates based on bottom-up inventories and top-down approaches lack consensus for specific regions, such as the U.S. Corn Belt for N_2_O^3−6^ and oil and gas fields for CH_4._^7−9^

Aircraft extensively serve as a valuable mobile platform for atmospheric observations, enabling the estimation of greenhouse gas (GHG) emissions from local to regional scales. A mass balance approach has been commonly used with airborne CH_4_ measurements^7,10−13^ but less used for N_2_O^14,15^ due to its typically small enhancements over the background. The development of a mid-infrared absorption spectrometer in the past decade made it feasible for high-precision airborne N_2_O measurements, thereby improving the signal-to-noise ratio of N_2_O and allowing for the application of a mass balance approach to CH_4_ and N_2_O. Recently, Gvakharia et al. (2020)^14^ applied the mass balance approach to quantify N_2_O emissions from fertilizer plants and fertilized croplands. Similarly, Herrera et al. (2021)^15^ employed a boundary layer budget approach with airborne observations to estimate N_2_O emissions from agricultural and urban areas.

Performing high-precision continuous airborne measurements, especially for N_2_O, poses challenges. To date, airborne measurements of N_2_O have been achieved simultaneously with other trace gases like CH_4_ by quantum cascade laser spectrometers (QCLSs) in the campaigns such as CalNex,^16^ FEAST,^17^ ACT-America,^6,18^ the NASA DISCOVER-AQ mission^15^ in the U.S. and GAUGE and MAMM^19^ in England. During the performance test of an onboard QCLS, the retrieved concentrations of N_2_O and CH_4_ from optical spectrometers are severely affected by variations in cabin pressure,^17,19^ and several calibration strategies have been developed to correct for this issue but with their own limitations.^17−19^ Fluctuations in input sample pressure propagating to a QCLS’s cell were found to impact N_2_O measurements more prominently than other trace gases.^20^ Additionally, the limited space and available power, as well as weight constraints of the packed instrumentation systems,^18^ must be considered when using lightweight aircraft.

We have developed a lightweight and self-powered innovative atmospheric sampling system that enables continuous sampling of ambient air during flight, and with subsequent analyses of the collected air samples, we can retrieve atmospheric trace gas mole fractions along the flight trajectories. This sampler is based on the unmanned aerial vehicle (UAV) version of the active AirCore.^21^ Unlike the previous UAV version, our system can sample air even when there are significant ambient pressure changes and is suitable for long-distance flights due to its larger sampling volume. Our sampling system operates without calibration issues and is easily deployable on a lightweight aircraft. The collected air samples were analyzed by either two cavity ring-down spectroscopy (CRDS) analyzers in series or a QCLS system to obtain simultaneous measurements of N_2_O and CH_4_. Based on the airborne measurements, we applied a mass balance approach to estimate the emissions of N_2_O and CH_4_ from urban areas in the Netherlands and compared them with the Dutch inventory.

## 2 Materials and Methods

We have developed a novel active AirCore sampling system, which is capable of collecting air samples during flight for high-precision continuous mole fraction measurements of N_2_O, CH_4,_ CO_2_, and CO. The basic principle of the AirCore system is the same as the earlier UAV version,^21^ i.e., molecular diffusion of air samples in a long piece of tube is relatively slow so that mole fractions of trace gases along the flight track can be retrieved. Compared to the UAV version, the new active AirCore system has been improved in two aspects: (1) the sampling flow rate is regulated by a pump and a mass flow controller to handle the possibly drastic change of ambient pressure during ascent/descent, and (2) the AirCore tube is pressurized to increase the amount of collected air samples and the flight sampling duration accordingly.

To evaluate the AirCore sampling system, it was deployed on a lightweight aircraft, SkyArrow 650 TCNS ERA (operated by Wageningen University & Research), together with an in situ CO_2_ and CH_4_ analyzer (LI-COR Biosciences, type LI-7810, Lincoln, NE). A total of 13 flights have been made in the urban areas of Groningen, Utrecht, and Rotterdam in the Netherlands. In the following sections, we describe the design of the active AirCore sampling system, the apparatus of air sample analysis, the retrieval algorithm and the validation of the AirCore measurements, and the mass balance approach for estimates of urban emissions of both N_2_O and CH_4_.

### 2.1 AirCore Sampling System

**Figure 1:**
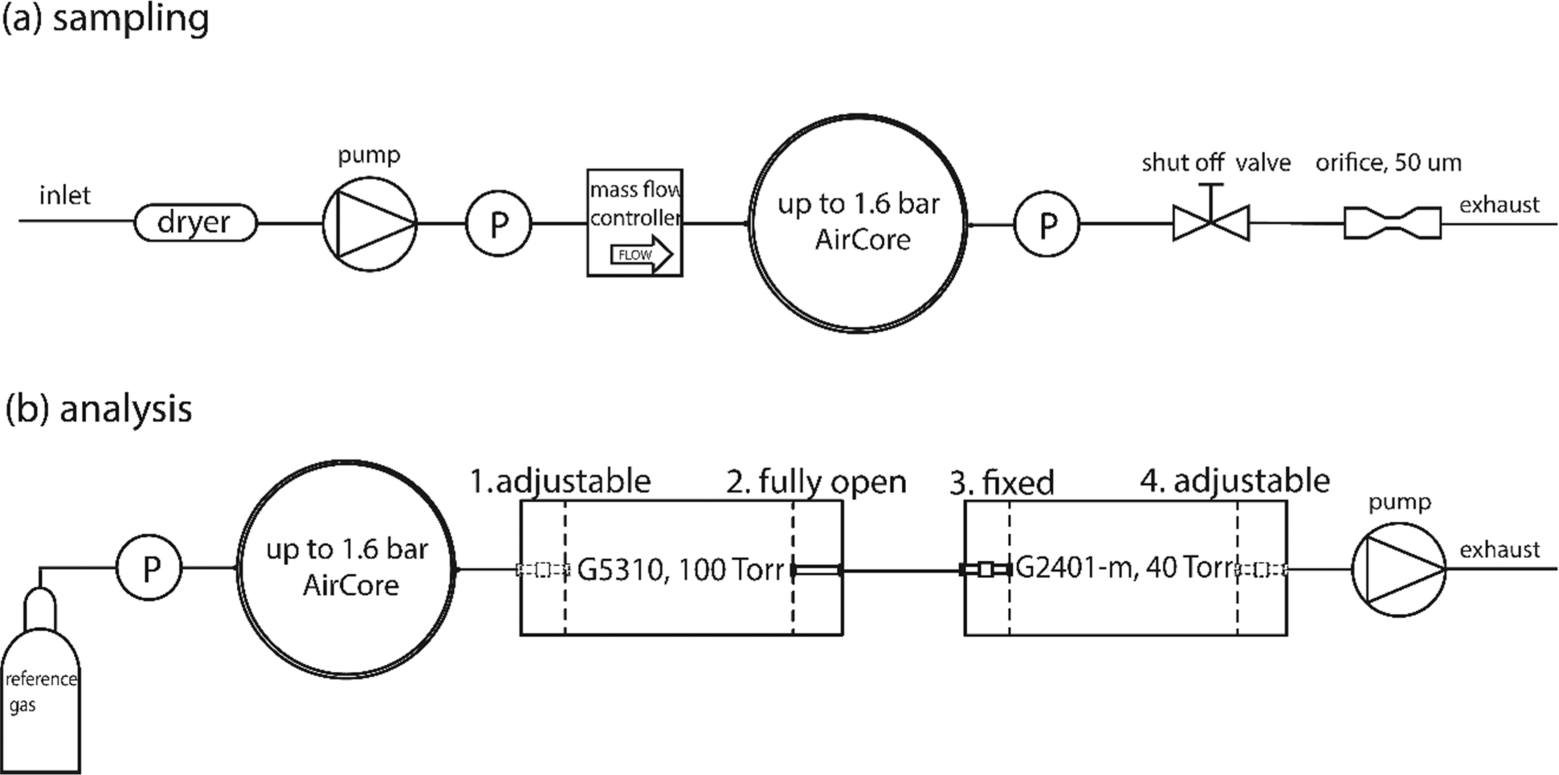
Flow diagrams of the active AirCore system: (a) for the AirCore sampling and (b) for the AirCore sample analysis. The air samples are analyzed using two Picarro analyzers connected in series. Each analyzer has two proportional valves, one at the inlet and one at the outlet. Of these, 1 and 4 are controlled using the analyzer software (i.e., inlet control mode for the G5310 and outlet control mode for the G2401-m), 2 is set to be fully open, and 3 is manually set to a fixed value. Alternatively, analyzers (e.g., a single QCLS system) may be employed instead of the dual-Picarro setup depicted here.

The design of the AirCore sampling system is shown in Figure 1a. The essential point is to maintain a constant sample mass flow rate throughout the flight, delivered here using a diaphragm pump (KNF NMP015 KPDC-B 6 V) and a mass flow controller (a Bronkhorst IQFlow-200C-AAD-11–V-S). The AirCore coil is made of a ∼285 m stainless steel tube 3/16”OD, with its inner surface coated with SilcoNert 1000 (SilcoTek) (other parameters are shown in Table S1). The collected air sample is dried using a chemical dryer filled with magnesium perchlorate located at the inlet of the system. Two pressure sensors of the same type (AmSys AMS 5915–2000-A-3 V) are installed: one between the pump and the mass flow controller to diagnose the performance of the pump and one at the outlet of the AirCore tube to indicate the pressure of the collected air sample inside the coil. An orifice (50 μm) is placed at the outlet of the AirCore system to limit the flow out of the AirCore coil, which effectively builds up the pressure inside the AirCore coil and prevents (humid) air from flowing into the AirCore from the outlet side in the case that ambient pressure increases rapidly (e.g., during descent). Before flight, the AirCore is filled with a dry calibration gas (hereafter termed the fill gas). During flight sampling, the AirCore system functions in two different modes: (1) flow-through: the fill gas is slowly squeezed out of the coil through the orifice, and the out-flow rate depends on the pressure gradient across the orifice connecting to the ambient atmosphere; (2) pressurizing: when ∼93% of the fill gas has flown out of the coil, a shut-off valve located upstream of the orifice will be closed, and from this point onward, the AirCore will be pressurized until it reaches the highest allowed pressure of ∼1.6 bar (Figure S1). Without the orifice, the pressure of the air sample inside the AirCore would be equal to the ambient pressure during the flow-through mode, while the pressure upper limit of ∼1.6 bar was chosen to avoid possible damage of the inlet valve of the Picarro analyzer. All of the components are contained in a foam box (50 × 30 × 10 cm). Also included is a six-port two-position switching valve (not drawn) that avoids the need of taking the coil out of the box during gas filling, sampling, storage, and analysis.

### 2.2 Sample Analysis

#### 2.2.1 Two Picarro CRDS in Series

To measure the concentrations of both N_2_O and CH_4_, a Picarro N_2_O/CO/H_2_O analyzer (model G5310) and a Picarro CO_2_/CH_4_/CO/H_2_O analyzer (model G2401-m) were used, thus providing measurements of CO_2_ and CO as well. The two Picarro analyzers were configured in series (Figure 1b) in such a way that a critical flow is obtained via the inlet proportional valve of the second analyzer. As the cavity pressure of the G5310 and the G2401-m was maintained at 133 hPa (100 Torr) and 53 hPa (40 Torr), respectively, and the outlet proportional valve of the G5310 was fully open, the flow rate through the inlet proportional valve of the G2401-m was critical and constant. The analysis flow rate could be manually adjusted with the inlet valve of the G2401-m and was set to various values between analyses (but constant for any single analysis), ranging from ∼47.5 sccm (standard cubic centimeters per minute) to ∼66.7 sccm. The inlet valve of the G5310 and the outlet valve of the G2401-m were controlled using the analyzer software to maintain constant cavity pressures. Prior to sample analysis, a reference gas flows through the analyzers (bypassing the AirCore) for ∼20 min to flush the sampling line. The pressure of the reference gas is adjusted to the same value of the AirCore coil pressure, e.g., 1.6 bar.

Under this configuration, for all of the flights, the precision (1σ at 0.5 Hz) of the G5310 measurements was <0.12 and <0.2 ppb for N_2_O and for CO, respectively; the precision (1σ at 0.25 Hz) of the G2401-m was ≤300, 1, and 7 ppb for CO_2_, CH_4_, and CO, respectively. The physical cell volume of G5310 and G2401-m is 48 mL (maintained at 133 hPa or 100 Torr and 40 °C) and 35 mL (maintained at 53 hPa or 40 Torr and 45 °C), respectively. The equivalent cell volume at the standard temperature and pressure (i.e., STP, 273.15 K and 1013.25 hPa) of the G5310 and the G2401-m is about 5.5 and 1.6 mL, respectively. This leads to the 63% response time of 6.6 and 1.9 s for the G5310 and the G2401-m at a typical analysis flow rate of 50 sccm.

#### 2.2.2 Aerodyne QCLS

For several flights, the sampled air was analyzed using a dual-laser QCLS (Aerodyne Inc.) for mole fraction measurements of N_2_O, CH_4_, CO, CO_2_, COS, and H_2_O. The first laser of the QCLS measured COS, CO_2_, CO, and H_2_O, and the second laser measured N_2_O, CH_4_, and H_2_O. The geometric cell volume of the QCLS is 0.15 L. The cell temperature was a constant 25 °C, and pressure was ∼53 hPa (40 Torr) in 2021 or ∼67 hPa (50 Torr) in 2022. This corresponds to the equivalent volume at STP of 7.2 and 9.1 mL. The analysis flow rate is constant, ranging from ∼46 to 64 sccm for the flights. The precision of the QCLS (1σ at 1 Hz) is better than 0.12, 0.6 ppb, 20 ppt, 0.2 ppm, 1 ppb, and 20 ppm for N_2_O, CH_4_, COS, CO_2_, CO, and H_2_O, respectively.

### 2.3 In Situ Observations

A total of 13 flights were made with the active AirCore system aboard the SkyArrow aircraft in 2020, 2021, and 2022 (Table S2). For 10 out of 13 flights, an in situ LI-7810 CH_4_/CO_2_/H_2_O trace gas analyzer (LI-COR Biosciences, Lincoln) was operated successfully next to the active AirCore system. The LI-7810 provides continuous measurements of CH_4_, CO_2_ and H_2_O. However, CO_2_ is measured as an ancillary species with lower precision. For all flights, the air samples were dried with a chemical dryer filled with magnesium perchlorate. With a physical cavity volume of 6.41 mL^3^ operating at a pressure of 400 hPa and a temperature of 55 °C, the equivalent cavity volume is 2.1 mL at STP, leading to a response time of ∼0.5 s at a typical flow rate of 250 sccm. Notice that the measurements were reported every second, i.e., at 1 Hz, which is slower than the response time of the analyzer.

A time lag of ∼14 s (from air entering the inlet until it reaches the cavity of the LI-7810 analyzer) was estimated by breathing to the inlet and checking the timing of the resulting CO_2_ peak before a flight. An LI-7810 analyzer was evaluated to be linear and shown in the ICOS Atmospheric Thematic Centre Test Reports for the LI-7810.^22^ We did not test the linearity of the LI-7810 analyzer flown during our flights by ourselves and assumed that the LI-7810 of the same type would perform similarly and would be linear. Therefore, we applied an offset correction based on measurements of one calibration gas before and after flights when available; the calibration gas traceable to the WMO scales was either with 2071.49 ppb CH_4_ and 434.27 ppm of CO_2_ or with 2013.7 ppb CH_4_ and 402.75 ppm of CO_2_. The drift before and after a flight was <0.7 ppb for CH_4_ and <7 ppm for CO_2_, respectively (Figure S2). The flights typically lasted ∼2.5 h with an average flying speed of ∼40 m/s and were made in the urban areas that surrounded either the city of Groningen, the city of Utrecht, or the city of Rotterdam in the Netherlands. For all flights, at least one vertical profile up to 1500–2000 m was made to determine the planetary boundary layer (PBL) height. Most flights took off in the late morning or early afternoon, and the specific information on the flights is shown in Table S2. Ambient meteorological parameters, such as air temperature and pressure, relative humidity, and wind speed and direction, were measured at 1 Hz using the sensors deployed on the SkyArrow during flights.^23^

### 2.4 Spatial Resolution of the AirCore Measurements

Similar to Andersen et al. 2018,^21^ the spatial resolution of the active AirCore measurements is mainly determined by (1) the molecular and the Taylor dispersion during sample collection, storage, and analysis; (2) the smearing effect caused by air mixing in the cavity of the Picarro analyzers or the QCLS during sample analysis; (3) the AirCore sampling flow rate; and (4) the aircraft flight speed.

We derive the diffusion length based on an effective diffusion coefficient in air that combines both the molecular diffusion and the Taylor dispersion^24^ and then calculate the diffusion volume at STP, Δ*V*_diff_, based on the diffusion length, the coil tube inner diameter, and the coil pressure. The calculated Δ*V*_diff_ varies among different species as the effective diffusion coefficient of each species is distinct (Table S3&S2.4.1).

The smearing effect was caused by the sample air mixing in the analyzer cell. During Picarro analysis, smearing happens once for N_2_O and CO which were measured using the first analyzer but twice for CH_4_, CO_2_, and CO which were measured using the second analyzer, while during QCLS analysis, the smearing effect happened once for all gases. Both the equivalent cell volume at STP and the volume for every measurement should be taken into account, and the smearing volume, Δ*V*_smear_, is defined as the larger one of them (Table S4&S2.4.2).

Taken together, a composite volume is calculated by summing the diffusion volume and smearing volume in quadrature. Molecular diffusion has a larger impact on the composite volume than the smearing effect does. Spatial resolution can be calculated as the product of the time that takes the AirCore to collect the composite volume and the flight speed of the SkyArrow. Regarding Picarro analysis, the spatial resolution for N_2_O and CO is  and for CH_4_, CO_2_, and CO is , while regarding QCLS analysis, the equation is the same for all gases: , where *f* is the sampling rate of the AirCore and *v* is the flying speed of the skyarrow plane. The spatial resolution ranges from 1.1 to 1.8 km and does not significantly vary among multiple gases but does vary among different flights due to variable sampling rate and flying speed (Table S5). The flying speed during vertical profiling is a bit lower than the horizontal flying speed, leading to a higher spatial resolution for vertical profiling.

### 2.5 Retrieval of AirCore Flight Measurements

To project AirCore trace gas concentrations onto the flight trajectories, we need to link the concentration measurements using the two Picarro analyzers or QCLS, reported as a function of the sample analysis time, with the flight coordinates and altitude, reported as a function of the GPS time. This is realized by linking the amount of sampled air molecules with the corresponding amount of analyzed air molecules. Since both the sampling and the analysis mass flow rates are constant, we use the fractions of the sampling and analysis time to establish the linkage.

**Figure 2:**
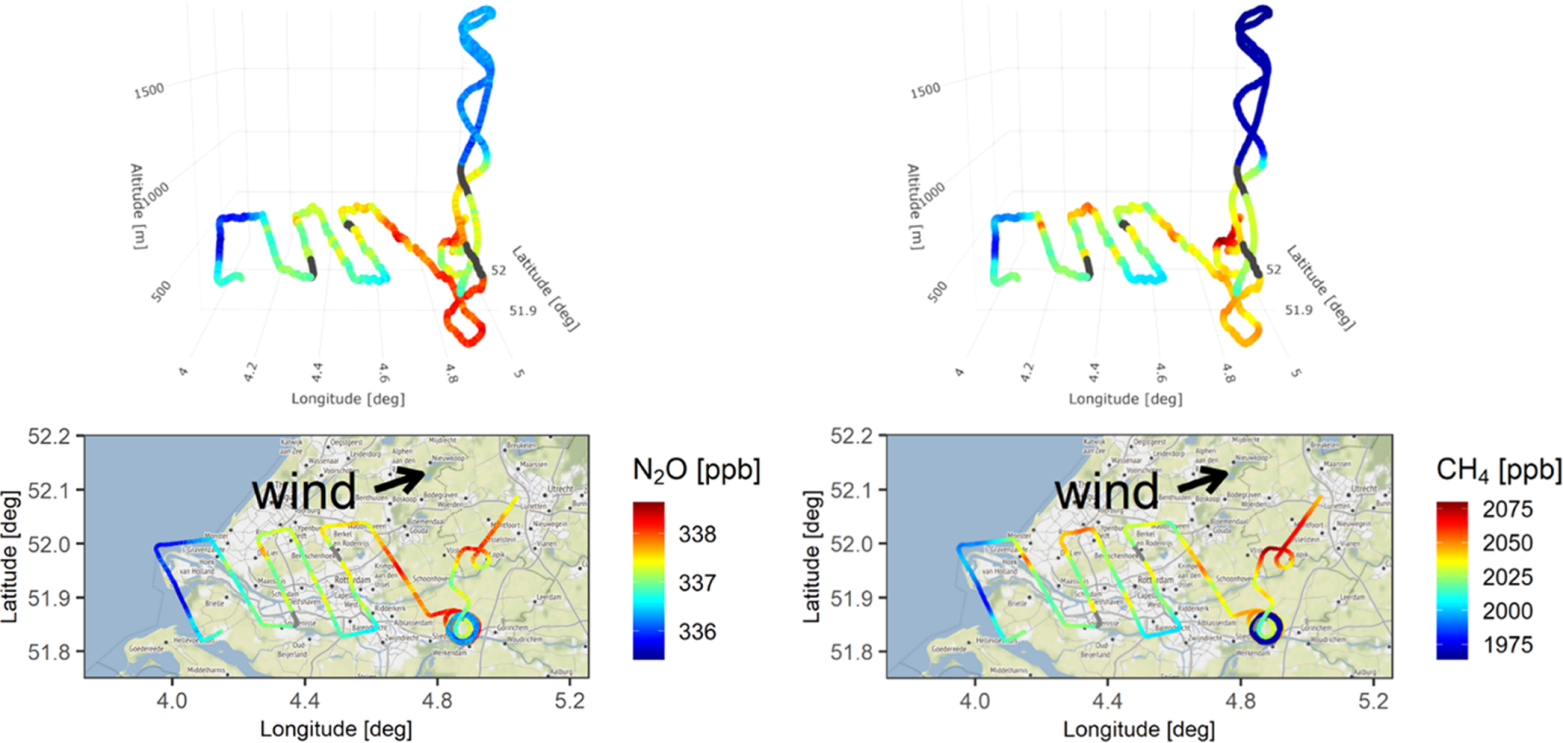
3D flight track and 2D map of retrieved AirCore measurements of mole fractions for N_2_O (left column) and CH_4_ (right column) for flight 0906. The base map is made using map tiles by Stamen Design (CC BY 3.0) and geographic data from OpenStreetMap (ODbL).

After flight, the AirCore typically contains approximately 5–7% fill gas. This fill gas is pulled into the analyzer ahead of the actual sample. As a first guess, the start of the actual sample is taken to be the midpoint of the CH_4_ concentration transition from fill gas to sample gas. Similarly, the end of the actual sample is defined by the midpoint of the transition from the sample to reference gas concentrations (Figure S3). The in situ CH_4_ measurements provide useful information to optimize the retrieval algorithm of the active AirCore system. A series of retrieval scenarios with different combinations of the chosen start and end points of the G2401-m/QCLS measurements were implemented. For each combination, the retrieved AirCore measurements were compared to the LI-7810 CH_4_ measurements. The LI-7810 CH_4_ measurements were first smoothed to roughly the same resolution of the AirCore measurements, and the maximum correlation between the two measurement time series indicated optimal retrieval (S2.5). The optimal start and end points are determined uniquely for QCLS measurements since the multiple gas concentrations are provided simultaneously as a function of the time but are different for two Picarro analyzer measurements. We determined the time difference between the two Picarro measurements of the AirCore samples using the common CO measurements from both analyzers. Then, we shifted time stamps of the start and end points of the G2401-m measurements to obtain the start and end points of the G5310 measurements. As an example, Figure 2 shows the retrieved AirCore concentration measurements for flight 0906.

### 2.6 Emission Estimation

The concurrent measurements of trace gas mole fractions and meteorological parameters allow us to estimate the surface emissions of trace gases using a mass balance approach. The enhancement plume of a species of interest relative to its background mole fraction is integrated across the width of the plume and the height of PBL. One flight over each of the three urban areas of Groningen (Figure S16), Utrecht (Figure S17), and Rotterdam (Figure 2) was selected to perform the mass balance estimation. Following the IG^3^IS guidelines,^25^ the PBL height should be determined from the vertical profiles of potential temperature (PT), which was indeed possible for several flights. When there was not a clear transition from the PBL to the free troposphere using the potential temperature, the PBL height was estimated based on the vertical profiles of N_2_O, CH_4_, CO_2_, CO, and water vapor (Table S2). The estimates of N_2_O and CH_4_ emissions for Groningen and Utrecht are determined as the difference of in-flow and out-flow fluxes (Table S7), while the presented Rotterdam emission estimates are derived using the mean mole fraction concentration of upwind transects as the background (S2.6). We measured the mole fractions of N_2_O, CH_4_, CO_2_, and CO for the AirCore samples, and in principle we can also estimate the emissions of CO_2_ and CO using the same approach. However, the emission estimates of CO_2_ and CO were not included because the discussion of the distinct sources and sinks of CO_2_ and CO from those of N_2_O and CH_4_ would distract from the main topic of this work. The comprehensive uncertainty of flux estimates for Utrecht and Rotterdam sums the uncertainties of mole fraction enhancements, wind speed, wind direction, plume width, and PBL height in quadrature, while plume width uncertainty was not considered for the Groningen flight because the plume width used in the equation is smaller than the real value due to the short flight transect (Table S9). Supporting Information (S2.6) provides details on the calculation of mass balance fluxes and their uncertainties.

### 2.7 Inventory Emissions

A 5 × 5 km^2^ grid map inventory developed by the Dutch government (https://data.emissieregistratie.nl/emissies/kaart?s=snD1oDkQH) was used to compare with the estimated mass balance fluxes. The sum of the emissions of the grids within the flight track was determined to be comparable for three urban areas, although there are concerns. In the case of Rotterdam, with the upwind as the background, it is appropriate to consider that the estimated mass balance fluxes come from the areas bounded by the flight tracks, assuming that there is no net transport of emissions through the flight tracks that are almost parallel to the wind direction. In the case of Groningen, the footprints of estimated fluxes are complicated to derive. The 1 min averaged wind (Figure S16) along different transects in the rectangular flight route show that the wind came from northeast, east, and southeast. And, the wind direction changed with altitude from northeast on the ground level to southeast at ∼1000 m agl (Figure S8). As a result, the plume observed on west transects may have been influenced by surface emissions from both the city center and south suburb areas. Regarding the whole flight track, the enhancements are observed on the west and south transects, and for the north and east transects, the mole fractions of N_2_O and CH_4_ are similarly low and can be determined to be background (Figure S10). Hence, the difference of out-flow fluxes (west transects) and in-flow fluxes (south transects) roughly came from the areas bounded by the flight tracks. In the case of Utrecht, the estimated fluxes should be comparable to the difference of the emissions that cause downwind background enhancements and downwind plume enhancements. However, the spatial resolution (5 × 5 km^2^) of the Dutch inventory is too low to separate the areas. There are ∼10 grids within the flight track, and following the wind direction, the grids are arranged diagonally (Figure S14). In this case, we do not think it is possible to separate the two types of emissions. What we can do is to robustly sum up the emissions of the grids within the flight track to compare with the mass balance estimates.

## 3 Results and Discussion

### 3.1 Comparison of the AirCore Measurements and LI-COR

**Figure 3:**
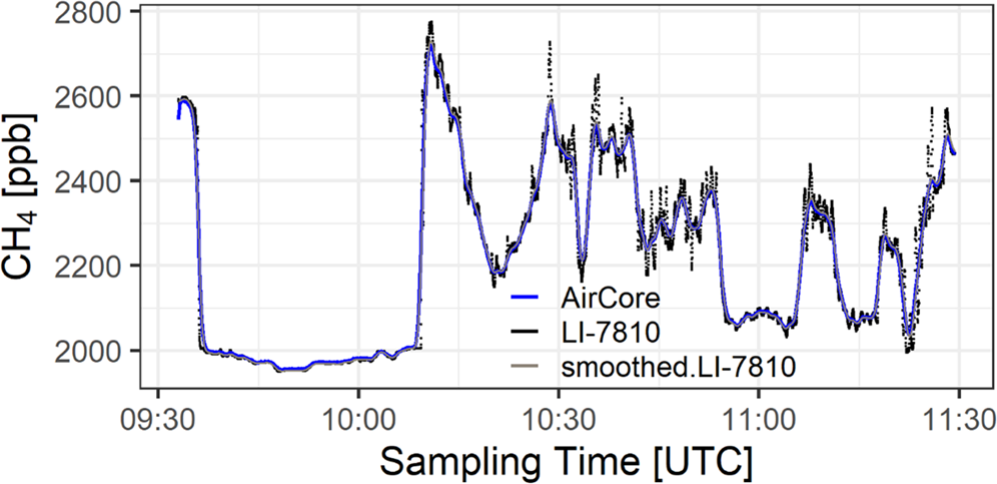
Comparison of AirCore and LI-7810 measurements for CH_4_.

AirCore measurements of CH_4_ are in good agreement with the smoothed LI-7810 measurements. As an example, the time series of the AirCore and the LI-7810 measurements of CH_4_ after optimal retrieval for flight 0915 is shown in Figure 3. The comparison for the other flights performed similarly; the correlation coefficient between the AirCore and the LI-7810 measurements ranged from 0.998 to 0.9999 and the RMSE between them ranged from 1.9 to 5.6 ppb among all of the flights (Table S6).

The AirCore measurements potentially under-represent small-scale mole fraction variations due to its lower spatiotemporal resolution compared to LI-7810. On the other hand, LI-7810 measurements occasionally become noisy. This is related to the drastic ambient pressure drop, which causes an unidentified change in the LI-COR behavior that is insufficiently compensated for in LI-COR software. AirCore sampling has three advantages for airborne measurements: (1) easy flight operation, (2) suitable for deployment on a lightweight aircraft due to its compact size and low power requirement, and (3) simultaneous high-precision atmospheric mole fraction measurements of multiple species.

### 3.2 Enhancements of N_2_O and CH_4_ Downwind of Three Urban Areas

To perform mass balance flux estimation, three individual flights were selected over each of the urban areas of Groningen, Utrecht, and Rotterdam (for details, please see S2.6). For the flight over Rotterdam, we found that enhancements of N_2_O and CH_4_ relative to the upwind background are significantly higher than the uncertainty of the background (Table 1). This was not the case for the flights over Groningen and Utrecht. For the two flights, the enhancements relative to the upwind background are very small, even within the uncertainty of the background (Table S8). That is because both upwind and downwind transects show similarly clear plumes (Figure S13). We do not think that such small enhancements can be defined to be reliable to perform mass balance calculation. Furthermore, we cannot simply use the enhancements relative to the low concentrations of both edges of downwind transects to estimate the emissions from the urban areas of Groningen and Utrecht. These enhancements are caused most likely by emissions from the areas beyond the targeted urban areas.

**Table 1:** Enhancements (ppb) of the Three Urban Areas with Different Background Selection

city	background selection	N_2_O background	N_2_O enhancements^d^	CH_4_ background	CH_4_ enhancements^d^
Groningen	single edge of downwind (out-flow)^a^	336.7 ± 0.1	0.5 ± 0.2	2016 ± 1	19 ± 2
	single edge of upwind (in-flow)^a^	336.6 ± 0.1	0.4 ± 0.2	2009 ± 1	16 ± 2
Utrecht	both edges of downwind (out-flow)^b^	338.1 ± 0.1	0.5 ± 0.2	2124 ± 1	28 ± 2
	both edges of upwind (in-flow)^b^	338.1 ± 0.1	0.5 ± 0.2	2112 ± 3	21 ± 3
Rotterdam	upwind transect^c^	336.9 ± 0.2	0.9 ± 0.2	2019 ± 2	17 ± 2
	both edges of downwind^b^	337.7 ± 0.1	0.3 ± 0.2	2030 ± 2	12 ± 2

a Mean concentration of single edge of the downwind/upwind transect as the background: the uncertainty is represented by the combination of the variability of background (sd) and the measurement precision. Only one edge of downwind/upwind transects shows stable and low concentrations over Groningen because the flight track did not frame the enhancement plume.

b Linear function derived from both edges of the downwind/upwind transect as the background due to the concentration gradient between two edges: the uncertainty of background is represented by the systematic uncertainty and the measurement precision. The systematic uncertainty is the standard deviation of the residuals between the modeled values from the linear function and observed values.

c Mean concentration of the upwind transect as the background: the uncertainty is represented by the combination of the variability of background (sd) and the measurement precision.

d The average enhancements and their uncertainties. The uncertainties are derived by summing the background uncertainty (real numbers rather than the rounded numbers shown in the table) and the plume uncertainty in quadrature. The plume uncertainty is represented by the measurement precision, 0.1 ppb for N_2_O and 1 ppb for CH_4_.

Alternatively, we used the difference between the “in-flow” and “out-flow” flux to determine the emissions from the Groningen and Utrecht urban areas. The “in-flow” and “out-flow” fluxes were derived separately by using the up- and downwind transect enhancements relative to their edges (Table S7). For Groningen, the PBL was stationary (S2.6) during the flight, and the same PBL height was used for the “in-flow” and “out-flow” flux calculation, while the plume width and the perpendicular wind speed were different for the upwind and downwind transects. Time difference between the upwind and downwind transects was around 50 min, which may result in a significant difference in the PBL heights and thus affects the observed enhancements, especially when the emissions are relatively small. This is the case for Utrecht, where the PBL height increased from 650 to 750 m between the ascent and descent flights. The estimated urban area emissions are shown and discussed in section 3.3.

Based on vertical profiles, N_2_O mole fractions within the PBL do not have significant correlation with altitude for Groningen and Utrecht but do for Rotterdam, while CH_4_ mole fractions within the PBL significantly correlated with altitude (r-squared value >0.7) for the three urban areas (Figure S7). As for Rotterdam, the vertical profile was conducted downwind; therefore, we assumed that the downwind plume followed the same vertical distribution as the vertical profile, and upwind background was vertically well mixed. In addition, we also calculated the emissions assuming vertically well-mixed downwind plumes, which are ∼4% for N_2_O and ∼7% for CH_4_ larger than the estimates for not well-mixed plume. As for Utrecht, quick dynamic vertical mixing and the lack of vertical profile below ∼300 m hinder the usage of vertical profiles to estimate upwind and downwind plumes. In the case of Groningen, out-flow fluxes were derived from downwind transects at three altitudes, without the assumption of a well-mixed boundary layer. The vertical profile was conducted at background locations rather than near downwind plume locations and therefore cannot offer much useful information about the vertical distribution of downwind CH_4_ plumes. More details are presented in Supporting Information S2.6.4.

The mass balance approach faces a challenge in obtaining an enhancement that is detectable for urban areas, particularly for N_2_O. An enhancement is defined to be detectable if it exceeds the background uncertainty and the threshold has been determined to be 0.1–0.2 ppb for N_2_O and 1–3 ppb for CH_4_ for all flights. We assume an ideal situation to perform mass balance estimation in which the PBL is stable and fully mixed, the wind speed and direction are constant, the plume for targeted areas is isolated with the plume for other areas, and designed flight transects are perpendicular to the wind direction. If the emissions and background are constant, the three main factors that influence the enhancements are the (1) PBL height, (2) plume width, and (3) wind speed. Given variable combinations of the three factors (Table S10), we derived the minimum emissions that can be detected in the enhancements for determination of N_2_O and CH_4_ fluxes using the mass balance approach. The minimum detectable emissions have taken into account the background atmospheric variabilities and the analytical precision of our atmospheric observations. More details are shown in Supporting Information S3.2. The detection limit of CH_4_ from offshore gas facilities derived by a mass balance approach has been discussed recently, in which the detection level resulted from the maximum uncertainty of all parameters participating in flux calculation without considering varying meteorological conditions during the course of the flights.^26^

The minimum detectable emissions are shown in a range (Table S11) for the three urban areas, and the different ranges are caused by variations in the meteorological conditions in real situations. We have found that the estimated mass balance fluxes of N_2_O for Groningen and Utrecht are within the range of detectable minimum N_2_O emissions, while those for Rotterdam are above. For CH_4_, the mass balance estimates for all three urban areas are above the detectable minimum emissions. In the case of Groningen and Utrecht for N_2_O, the change of meteorological parameters in real situations may have led to a state that the emissions cannot be detectable in enhancements, which is consistent with the fact that we did not detect reliable N_2_O enhancements for “traditional” mass balance estimation and had to subtract in-flow fluxes from out-flow fluxes to represent the N_2_O emissions. In the case where the mass balance estimates are above the detectable emissions, the enhancements resulting from the targeted areas’ emissions can be detected, which is the case for Rotterdam for N_2_O and the three urban areas for CH_4_.

### 3.3 N_2_O and CH_4_ Emission Estimates

**Figure 4:**
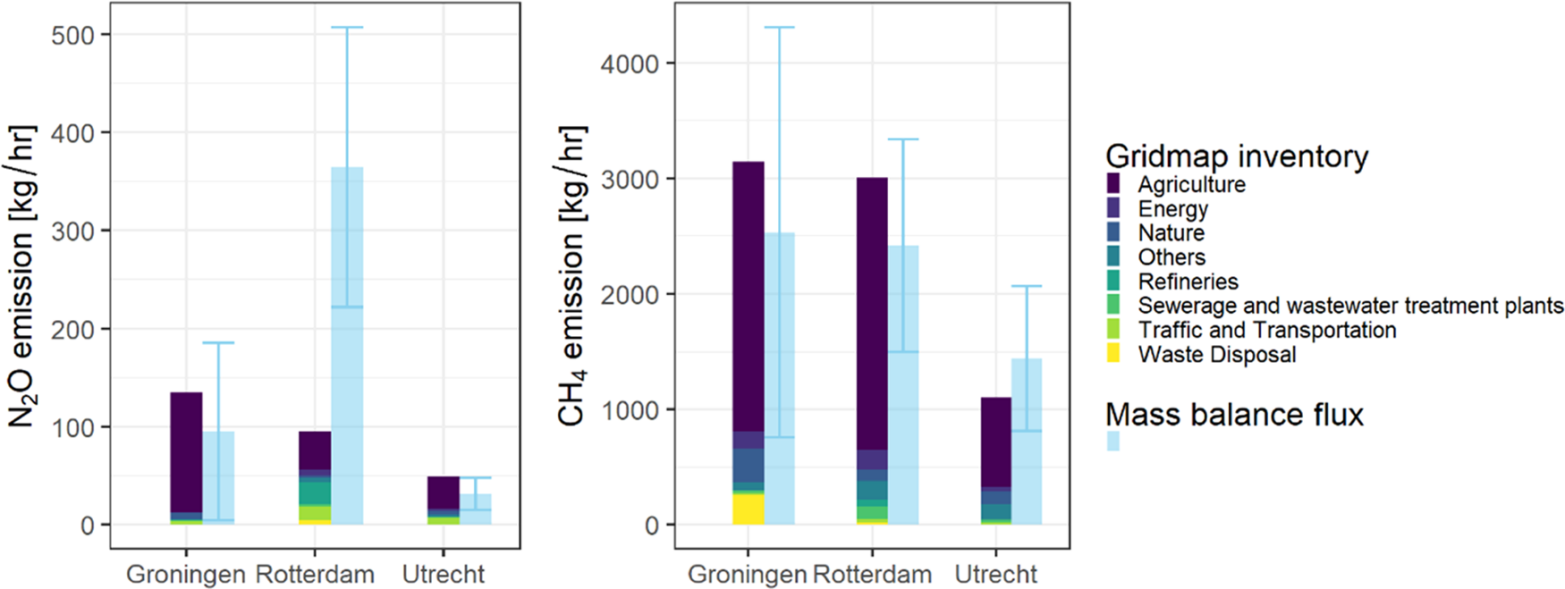
Comparison of mass balance fluxes and emission inventory-based estimates of Groningen, Rotterdam, and Utrecht for N_2_O and CH_4_. The mass balance fluxes are shown by the blue bar with uncertainties indicated by the error bars; the inventory-based estimates are shown by the stacked color bar, and different colors indicate the emissions from different sectors.

An overview of the estimates of N_2_O and CH_4_ emissions from three different urban areas is displayed in Figure 4, with their uncertainties and inventory-based estimates from different source sectors. The estimated N_2_O emissions for Rotterdam are several times larger than those of Groningen and Utrecht. The CH_4_ emission estimates for Groningen and Rotterdam are similar, and they are nearly two times larger than the estimate for Utrecht.

The relative uncertainty of the estimated emission from Groningen urban areas is the largest among those from the three urban areas (Figure 4 & Table S9). The relative uncertainties of in-flow and out-flow fluxes of Groningen and Utrecht are in the ranges of 33–78% for N_2_O and 28–63% for CH_4_, respectively. They are comparable to the uncertainties of the urban CH_4_ emissions estimated by the mass balance approach in previous studies.^12,27^ So far, urban N_2_O emissions have been rarely studied using the mass balance approach. Gvakharia et al. (2020)^14^ estimated the N_2_O emissions with the first application of the mass balance approach but focused on agricultural regions rather than urban areas. Herrera et al. (2021)^15^ used a boundary layer budget approach to derive both nocturnal and daytime emissions of N_2_O for several urban and agricultural areas in the U.S.; the relative uncertainty for daytime estimate is up to 95%, which is comparable to the uncertainty of Groningen’s N_2_O emission estimates, the largest among those of the three cities’ emission estimates.

Our estimated mass balance fluxes of N_2_O flux estimate for Rotterdam are several times larger than the inventory-based estimates, while our estimated CH_4_ fluxes are not significantly different from the Dutch inventory-based estimates, as is the case for N_2_O fluxes for Groningen and Utrecht (Figure 4). The inventory-based estimates were derived by summing up the grid map values of all of the grids that were within the flight tracks (Figure S14), which shows that agriculture is the largest source of both N_2_O and CH_4_ for the three urban areas (Figure 4). This is because the areas between the flight track and city boundary are mainly for agricultural usage. Considering the registered emissions within the boundary of each municipality, the shares of agricultural emissions in total emissions decrease and are not the largest anymore, except for agricultural N_2_O emissions from the municipality of Groningen, where they remain the largest (Figure S15). Yacovitch et al. (2018)^11^ quantified CH_4_ flux (8000 kg h^–1^) from the Groningen region using a mass balance approach and estimated emissions (14 000 kg h^–1^) based on inventory analysis; their mass balance flux is around 3 times larger than our estimate, while their study region is around 6 times larger than ours. Moreover, the Dutch 5 x 5 km^2^ inventory emission estimates represent yearly emissions, while our mass balance flux represents a snapshot estimate of emissions for a single day.

The exact reason that causes the underestimation of N_2_O emissions in the Rotterdam inventory compared to our estimated emissions is not known. One possible explanation is that N_2_O emissions from the wastewater treatment plant (WWTP) may be underestimated in the Dutch inventory, resulting in smaller total emissions for Rotterdam compared to the mass balance estimate. WWTP was previously recognized as a minor source for N_2_O in the 2006 IPCC guideline, but many studies have revealed that WWTP may emit substantial N_2_O.^28−30^ In the revised 2019 IPCC guidelines, an updated emission factor is presented to calculate WWTP emissions, which was also used in the Dutch inventory. The WWTP emission is highly uncertain due to specific process conditions and temperature. Therefore, emission estimates based on actual onsite measurements to quantify individual WWTP emissions, e.g., using mobile surveys or UAV measurements, are required to improve the emission factor and validate the inventory estimates.

In conclusion, our study demonstrates that aircraft-based AirCore sampling can be useful to estimate urban N_2_O and CH_4_ emissions combined with a mass balance approach. Further mass balance flights over different seasons are encouraged to reveal the temporal variation in urban N_2_O and CH_4_ emissions.

## Supporting Information Available

AirCore spatial resolution calculation, retrieval of AirCore flight measurements, mass balance flux calculation and its uncertainty estimation, and theoretical analysis of mass balance estimation (PDF)

## References

[ref1] Forster P.; Alterskjaer K.; Smith C.; Colman R.; Damon Matthews H.; Ramaswamy V.; Storelvmo T.; Armour K.; Collins W.; Dufresne J.; Frame D.; Lunt D.; Mauritsen T.; Watanabe M.; Wild M.; Zhang H.; Masson-Delmotte V.; Zhai P.; Pirani A.; Connors S.; Péan C.; Berger S.; Caud N.; Chen Y.; Goldfarb L.; Gomis M.; Huang M.; Leitzell K.; Lonnoy E.; Matthews J.; Maycock T.; Waterfield T.; Yelekçi O.; Yu R.; Zhou B. The Earth’s Energy Budget, Climate Feedbacks and Climate Sensitivity. In Climate Change 2021 – The Physical Science Basis; Cambridge University Press, 2023; pp 923–1054 10.1017/9781009157896.009.

[ref2] Ravishankara A. R.; Daniel J. S.; Portmann R. W. Nitrous Oxide (N2O): The Dominant Ozone-Depleting Substance Emitted in the 21st Century. Science 2009, 326 (5949), 123–125. 10.1126/science.1176985. 19713491

[ref3] Griffis T. J.; Lee X.; Baker J. M.; Russelle M. P.; Zhang X.; Venterea R.; Millet D. B. Reconciling the Differences between Top-down and Bottom-up Estimates of Nitrous Oxide Emissions for the U.S. Corn Belt. Global Biogeochem. Cycles 2013, 27, 746–754. 10.1002/gbc.20066.

[ref4] Fu C.; Lee X.; Griffis T. J.; Dlugokencky E. J.; Andrews A. E. Investigation of the N2O Emission Strength in the U. S. Corn Belt. Atmos. Res. 2017, 194 (January), 66–77. 10.1016/j.atmosres.2017.04.027.

[ref5] Chen Z.; Griffis T. J.; Millet D. B.; Wood J. D.; Lee X.; Baker J. M.; Xiao K.; Turner P. A.; Chen M.; Zobitz J.; Wells K. C. Partitioning N2O Emissions within the U.S. Corn Belt Using an Inverse Modeling Approach. Global Biogeochem. Cycles 2016, 30, 1192–1205. 10.1002/2015GB005313.

[ref6] Eckl M.; Roiger A.; Kostinek J.; Fiehn A.; Huntrieser H.; Knote C.; Barkley Z. R.; Ogle S. M.; Baier Bc.; Sweeney C.; Davis K. J. Quantifying Nitrous Oxide Emissions in the U.S. Midwest: A Top-Down Study Using High Resolution Airborne In-Situ Observations. Geophys. Res. Lett. 2021, 48 (5), e2020GL091266 10.1029/2020GL091266.

[ref7] Karion A.; Sweeney C.; Kort E. A.; Shepson P. B.; Brewer A.; Cambaliza M.; Conley S. A.; Davis K.; Deng A.; Hardesty M.; Herndon S. C.; Lauvaux T.; Lavoie T.; Lyon D.; Newberger T.; Pétron G.; Rella C.; Smith M.; Wolter S.; Yacovitch T. I.; Tans P. Aircraft-Based Estimate of Total Methane Emissions from the Barnett Shale Region. Environ. Sci. Technol. 2015, 49 (13), 8124–8131. 10.1021/acs.est.5b00217. 26148550

[ref8] Mehrotra S.; Faloona I.; Suard M.; Conley S.; Fischer M. L. Airborne Methane Emission Measurements for Selected Oil and Gas Facilities across California. Environ. Sci. Technol. 2017, 51 (21), 12981–12987. 10.1021/acs.est.7b03254. 29019666

[ref9] Francoeur C. B.; McDonald B. C.; Gilman J. B.; Zarzana K. J.; Dix B.; Brown S. S.; de Gouw J. A.; Frost G. J.; Li M.; McKeen S. A.; Peischl J.; Pollack I. B.; Ryerson T. B.; Thompson C.; Warneke C.; Trainer M. Quantifying Methane and Ozone Precursor Emissions from Oil and Gas Production Regions across the Contiguous US. Environ. Sci. Technol. 2021, 55 (13), 9129–9139. 10.1021/acs.est.0c07352. 34161066

[ref10] Tadić J. M.; Michalak A. M.; Iraci L.; Ilić V.; Biraud S. C.; Feldman D. R.; Bui T.; Johnson M. S.; Loewenstein M.; Jeong S.; Fischer M. L.; Yates E. L.; Ryoo J. M. Elliptic Cylinder Airborne Sampling and Geostatistical Mass Balance Approach for Quantifying Local Greenhouse Gas Emissions. Environ. Sci. Technol. 2017, 51 (17), 10012–10021. 10.1021/acs.est.7b03100. 28727429

[ref11] Yacovitch T. I.; Neininger B.; Herndon S. C.; van der Gon H. D.; Jonkers S.; Hulskotte J.; Roscioli J. R.; Zavala-Araiza D. Methane Emissions in the Netherlands: The Groningen Field. Elem. Sci. Anthropocene 2018, 6, 57 10.1525/elementa.308.

[ref12] Klausner T.; Mertens M.; Huntrieser H.; Galkowski M.; Kuhlmann G.; Baumann R.; Fiehn A.; Jöckel P.; Pühl M.; Roiger A. Urban Greenhouse Gas Emissions from the Berlin Area: A Case Study Using Airborne CO2 and CH4 in Situ Observations in Summer 2018. Elem. Sci. Anthropocene 2020, 8 (1), 15 10.1525/elementa.411.

[ref13] Fiehn A.; Kostinek J.; Eckl M.; Klausner T.; Galkowski M.; Chen J.; Gerbig C.; Röckmann T.; Maazallahi H.; Schmidt M.; Korbeń P.; Neçki J.; Jagoda P.; Wildmann N.; Mallaun C.; Bun R.; Nickl A. L.; Jöckel P.; Fix A.; Roiger A. Estimating CH4, CO2and CO Emissions from Coal Mining and Industrial Activities in the Upper Silesian Coal Basin Using an Aircraft-Based Mass Balance Approach. Atmos. Chem. Phys. 2020, 20 (21), 12675–12695. 10.5194/acp-20-12675-2020.

[ref14] Gvakharia A.; Kort E. A.; Smith M. L.; Conley S. Evaluating Cropland N 2 O Emissions and Fertilizer Plant Greenhouse Gas Emissions With Airborne Observations. J. Geophys. Res.: Atmos. 2020, 125 (16), e2020JD032815 10.1029/2020JD032815.

[ref15] Herrera S. A.; Diskin G. S.; Harward C.; Sachse G.; De Wekker S. F. J.; Yang M.; Choi Y.; Wisthaler A.; Mallia D. V.; Pusede S. E. Wintertime Nitrous Oxide Emissions in the San Joaquin Valley of California Estimated from Aircraft Observations. Environ. Sci. Technol. 2021, 55 (8), 4462–4473. 10.1021/acs.est.0c08418. 33759511

[ref16] Xiang B.; Miller S. M.; Kort E. A.; Santoni G. W.; Daube B. C.; Commane R.; Angevine W. M.; Ryerson T. B.; Trainer M. K.; Andrews A. E.; Nehrkorn T.; Tian H.; Wofsy S. C. Nitrous Oxide (N 2 O) Emissions from California Based on 2010 CalNex Airborne Measurements. J. Geophys. Res.: Atmos. 2013, 118 (7), 2809–2820. 10.1002/jgrd.50189.

[ref17] Gvakharia A.; Kort E. A.; Smith M. L.; Conley S. Testing and Evaluation of a New Airborne System for Continuous N2O, CO2, CO, and H2O Measurements: The Frequent Calibration High-Performance Airborne Observation System (FCHAOS). Atmos. Meas. Tech. 2018, 11 (11), 6059–6074. 10.5194/amt-11-6059-2018.

[ref18] Kostinek J.; Roiger A.; Davis K. J.; Sweeney C.; Digangi J. P.; Choi Y.; Baier B.; Hase F.; Groß J.; Eckl M.; Klausner T. Adaptation and Performance Assessment of a Quantum and Interband Cascade Laser Spectrometer for Simultaneous Airborne in Situ Observation of CH4, C2H6, CO2, CO and N2O. Atmos. Meas. Tech. Discuss. 2019, 12, 1767–1783. 10.5194/amt-12-1767-2019.

[ref19] Pitt J. R.; Le Breton M.; Allen G.; Percival C. J.; Gallagher M. W.; J-B Bauguitte S.; O’Shea S. J.; Muller J. B. A.; Zahniser M. S.; Pyle J.; Palmer P. I. The Development and Evaluation of Airborne in Situ N2O and CH4 Sampling Using a Quantum Cascade Laser Absorption Spectrometer (QCLAS). Atmos. Meas. Tech. 2016, 9 (1), 63–77. 10.5194/amt-9-63-2016.

[ref20] Santoni G. W.; Daube B. C.; Kort E. A.; Jiménez R.; Park S.; Pittman J. V.; Gottlieb E.; Xiang B.; Zahniser M. S.; Nelson D. D.; McManus J. B.; Peischl J.; Ryerson T. B.; Holloway J. S.; Andrews A. E.; Sweeney C.; Hall B.; Hintsa E. J.; Moore F. L.; Elkins J. W.; Hurst D. F.; Stephens B. B.; Bent J.; Wofsy S. C. Evaluation of the Airborne Quantum Cascade Laser Spectrometer (QCLS) Measurements of the Carbon and Greenhouse Gas Suite – CO2, CH4, N2O, and CO – during the CalNex and HIPPO Campaigns. Atmos. Meas. Tech. 2014, 7 (6), 1509–1526. 10.5194/amt-7-1509-2014.

[ref21] Andersen T.; Scheeren B.; Peters W.; Chen H. A UAV-Based Active AirCore System for Measurements of Greenhouse Gases. Atmos. Meas. Tech. 2018, 11 (5), 2683–2699. 10.5194/amt-11-2683-2018.

[ref22] Laboratory I. A. M. Evaluation Report for the LICOR LI-7810 Instrument, 2020.

[ref23] Vellinga O. S.; Dobosy R. J.; Dumas E. J.; Gioli B.; Elbers J. A.; Hutjes R. W. A. Calibration and Quality Assurance of Flux Observations from a Small Research Aircraft. J. Atmos. Oceans Technol. 2013, 30 (2), 161–181. 10.1175/JTECH-D-11-00138.1.

[ref24] Aris R. On the Dispersion of a Solute in a Fluid Flowing through a Tube. Proc. R. Soc. London, Ser. A 1956, 235 (1200), 67–77. 10.1098/rspa.1956.0065.

[ref25] Turnbull J.; DeCola P.; Mueller K.; Vogel F.; Karion A.; Lopez Coto I.; Whetstone J. IG3IS Urban Greenhouse Gas Emission Observation and Monitoring Best Research Practices; 2022; Vol. GAW Report. https://library.wmo.int/index.php?lvl=notice_display&id=22120#.YxC7-y2B2gT.

[ref26] Pühl M.; Roiger A.; Fiehn A.; Gorchov Negron A. M.; Kort E. A.; Schwietzke S.; Pisso I.; Foulds A.; Lee J.; France J. L.; Jones A. E.; Lowry D.; Fisher R. E.; Huang L.; Shaw J.; Bateson P.; Andrews S.; Young S.; Dominutti P.; Lachlan-Cope T.; Weiss A.; Allen G. Aircraft-Based Mass Balance Estimate of Methane Emissions from Offshore Gas Facilities in the Southern North Sea. Atmos. Chem. Phys. Discuss. 2023, 1–32. 10.5194/acp-2022-826.

[ref27] Heimburger A. M. F.; Harvey R. M.; Shepson P. B.; Stirm B. H.; Gore C.; Turnbull J.; Cambaliza M. O. L.; Salmon O. E.; Kerlo A.-E. M.; Lavoie T. N.; Davis K. J.; Lauvaux T.; Karion A.; Sweeney C.; Brewer W. A.; Hardesty R. M.; Gurney K. R. Assessing the Optimized Precision of the Aircraft Mass Balance Method for Measurement of Urban Greenhouse Gas Emission Rates through Averaging. Elem. Sci. Anthr. 2017, 5, 26 10.1525/elementa.134.

[ref28] Foley J.; de Haas D.; Yuan Z.; Lant P. Nitrous Oxide Generation in Full-Scale Biological Nutrient Removal Wastewater Treatment Plants. Water Res. 2010, 44 (3), 831–844. 10.1016/j.watres.2009.10.033. 19913869

[ref29] Gruber W.; von Känel L.; Vogt L.; Luck M.; Biolley L.; Feller K.; Moosmann A.; Krähenbühl N.; Kipf M.; Loosli R.; Vogel M.; Morgenroth E.; Braun D.; Joss A. Estimation of Countrywide N2O Emissions from Wastewater Treatment in Switzerland Using Long-Term Monitoring Data. Water Res. X 2021, 13 (September), 100122 10.1016/j.wroa.2021.100122. 34661091 PMC8503907

[ref30] Kosonen H.; Heinonen M.; Mikola A.; Haimi H.; Mulas M.; Corona F.; Vahala R. Nitrous Oxide Production at a Fully Covered Wastewater Treatment Plant: Results of a Long-Term Online Monitoring Campaign. Environ. Sci. Technol. 2016, 50 (11), 5547–5554. 10.1021/acs.est.5b04466. 27218458

