# Supplementary material for: Aircraft-Based AirCore Sampling for Estimates of N2O and CH4 Emissions
Source: Environ Sci Technol. 2023 Oct 5;57(41):15571–9. doi: 10.1021/acs.est.3c04932 (PMC10586372; doi:10.1021/acs.est.3c04932)
Supplement: Supplementary file 1 — es3c04932_si_001.pdf [file es3c04932_si_001.pdf]

Supporting Information (SI) for Research Article

Aircraft-based AirCore sampling for estimates of N_2O and CH_4 emissions

Xin Tong[1], Steven van Heuven[1], Bert Scheeren[1], Bert Kers[1], Ronald Hutjes[3], Huilin Chen*[1,2]

1 Centre for Isotope Research (CIO), Energy and Sustainability Research Institute Groningen (ESRIG), University of Groningen, 9747 AG, Groningen, The Netherlands

2 Joint International Research Laboratory of Atmospheric and Earth System Sciences, School of Atmospheric Sciences, Nanjing University, 210023, Nanjing, China

3 Water Systems and Global Change, Department of Environmental Sciences, Wageningen University and Research, 6708 PB, Wageningen, The Netherlands

Corresponding Author: Huilin Chen, email: Huilin.Chen@nju.edu.cn; Huilin.Chen@rug.nl

Number of pages: 37

Number of figures: 18

Number of tables: 11

2.4 The spatial resolution

2.4.1 Diffusion and Taylor dispersion

The molecular diffusion and Taylor dispersion both occurred during the sampling and storage of the AirCore, and are combined together to determine an effective diffusion coefficient. The sampling rate of the AirCore ranged from ~ 30 sccm to ~ 40 sccm for all flights, which meets the requirement of laminar flow in the AirCore tube. Hence, the effective diffusion coefficient can be calculated for a laminar flow profile inside the tube, by the equation as below,

$$D_{eff} = D + \frac{a^2\overline{V}^2}{48*D},$$

where D is diffusion coefficient for different gas species in the air near STP (D is 0.1436 for N_2O, 0.1952 $cm^2\ s^{-1}$ for CH_4, 0.1381 $cm^2\ s^{-1}$ for CO_2 and 0.1807 for CO; Massman, 1998), a is the inner radius of the AirCore tube, and $\overline{V}$ is the average velocity of the air inside the AirCore tube, which is, in our case, the ratio of the sampling rate and cross-sectional area.

The diffusion volume originating from the diffusion and Taylor dispersion is calculated as, $\Delta V_{diff} = 2 \cdot \sqrt{2 \cdot D_{eff} \cdot t} \cdot A$, where the factor 2 indicates the diffusion occurs forward in two directions, t is traveling time, which, in our case, is the time from the moment Skyarrow plane taking off to the start of concentration measurements, and A is the cross-sectional area of the AirCore tube.

2.4.2 Smearing effect

During analysis, the sampled air flowed through the analyzer cell and the volume in the cell mixed, leading to a smearing effect. Both the equivalent cell volume at STP and the volume for every measurement should be taken into account, and the smearing volume, ΔV_{smear}, is defined as the larger one of them. The volume for each measurement depends on both the measurement frequency and the flow rate, and the equivalent cell volume depends on the operating conditions. We listed the two types of the volumes for each flight (Table S4), and the ΔV_{smear} is the equivalent cell volume, the volume for each measurement, and the equivalent cell volume for the measurements performed by the Picarro G-5310 and G2401-m, and Aerodyne QCLS, respectively. Regarding the Picarro analysis, the smearing effect happened once for N_2O and CO that were measured by the first analyzer of the two Picarro analyzers in series, but twice for CH_4, CO_2, and CO which were measured by the second analyzer, while the smearing effect happened once for all gases regarding the QCLS analysis.

2.4.3 spatial resolution

Regarding the Picarro analysis, the spatial resolution for N_2O and CO is:

$$\Delta d = \sqrt{\Delta V_{diff}{}^2 + \Delta V_{smear-G5310}{}^2} \div f \times v,$$

and for CH_4, CO_2, and CO is $\Delta d = \sqrt{\Delta V_{diff}{}^2 + \Delta V_{smear-G5310}{}^2 + \Delta V_{smear-G2401}{}^2} \div f \times v$, and the equation is the same for all gases regarding QCLS analysis: $\Delta d = \sqrt{\Delta V_{diff}{}^2 + \Delta V_{smear-QCL}{}^2} \div f \times v$, where f is the sampling rate of the AirCore, and v is the flying speed of the skyarrow plane. The spatial resolution is unique for different gas species for each flight, and we listed the range of the spatial resolution for each gas species during all of the flights.

2.5 Retrieval of AirCore flight measurements

2.5.1 The optimization of retrieval by the validation of in-situ CH_4 measurements

The timestamps indicating the start and end of sampling period from the measured mole fraction profiles are determined from the validation of in-situ CH_4 measurements by a Licor LI-7810 CH_4/CO_2/H2O Trace Gas Analyzer.

With a first estimation of the start and end points, we selected a sampling period from the measured mole fraction profiles and switched them until the measured mole fraction matched well with the LI-7810 measurements.

The resolution of LI-7810 measurements is higher than that of the measurements of the aircraft-based active AirCore. Before the comparison of both, the CH_4 measurements of LI-7810 were smoothed firstly using Gaussian distribution to the same resolution of the AirCore measurements. The spatial resolution of the active AirCore measurements is related to storage time. Hence, we provided a range of the storage time based on landing time and the start time of analysis, resulting in a range of derived AirCore resolution. There is a parameter σ in the Gaussian smoothing function, equal to 0.5*AirCore resolution, and for each σ within the range, the CH_4 mole fraction from LI-7810 was smoothed and compared with the measured CH_4 from CRDS or QCLS analysers.

Following the basic rule that the number of moles of the sampled air stays constant, the flight information and the measured mole fraction profiles are linked together, following:

$$n = \Delta T_s \cdot f_s = \Delta T_m \cdot f_m$$

Since f_s and f_m are both constant values, the sampling time ΔT_s is also proportional to the measurement time ΔT_m. For each unit time, the measured mole fraction with analysis time is interpolated into the sampling time series. Hence, the measured AirCore mole fraction can be directly linked with the original flight time, GPS coordinates, and altitude. We use the 'the percent of the mole fraction transition' to define the start/end point of sampling; for the start, the mole fraction transits from the 'fill gas' to the 'sampled air', and for the end, the mole fraction transits from the 'sampled air' to the 'reference gas'. The start/end point was initially selected as the point when the percent of the CH_4 mole fraction transition was 50% and was optimally determined by the validation of in-situ CH_4 measurements of LI-7810. The Pearson correlation coefficient and root-mean-square error (RMSE) for the whole sampling time series was selected as an indicator to evaluate if the retrieval is optimal. As shown in Figure S3, the optimized AirCore retrieval is determined for September 15th with R^2=0.9999. The correlation coefficient and RMSE of in-situ CH_4 and optimized AirCore measurements for each flight is shown in the Table S6.

2.5.2 the optimization rule for the retrieval without the validation of in-situ measurements

The retrieval for the flight August 20th needs to be optimized by combining the other four flights in 2020 in the same campaign since there were no reliable in-situ measurements on that day. The range of the percentage of the transition is set as 55%-85% and 65%-95% for the start and end point, with an interval of 2%. For each combination of the start and end, we repeat the procedure described in the previous section 4.1, thereby generating a matrix of correlation coefficient with each combination for the other four flights. The combined correlation coefficient of the other four flights is determined as:

$$R^2 = \sqrt{\left(R_1{}^2\right)^2 + \left(R_2{}^2\right)^2 + \left(R_3{}^2\right)^2 + \left(R_4{}^2\right)^2},$$

in which, $R_1{}^2, R_2{}^2, R_3{}^2$, and $R_4{}^2$ are the correlation coefficients of the other four flights respectively. The combination of the start and end point was used to retrieve the AirCore measurements on August 20th when the combined correlation coefficient R^2 showed a maximum.

2.6 Emission estimation

2.6.1. The overview of the three flights to apply the mass balance approach

The flights 0601, 0917, and 0906 over Groningen, Utrecht, and Rotterdam, respectively, are selected to apply the mass balance approach to estimate urban emissions. The mass balance approach has been used widely to estimate the emissions from local to urban scales for CO_2, CH_4, and CO. In this study, we performed mass balance estimation on urban scales for N_2O and CH_4. The mass-balance estimation of N_2O emissions from urban areas is very limited.

$$flux = \int_{ground}^{PBL} \Delta c \cdot U_{\perp} \cdot dz \int_{-x}^{x} dx$$

The three flights have different patterns (Figure 2 & Figure S16 & Figure S17), and based on the following analysis, each flight has its own limitations to perform mass balance estimation. However, it is very challenging to achieve all of the ideal requirements to apply this approach, especially considering meteorological conditions in the Netherlands. Nevertheless, the estimated emissions based on this approach are still useful to evaluate the inventory-based estimates. In this section, we overviewed the flight patterns and the issues that are not ideal for the mass balance estimation. The more specific discussion to solve the issues are presented in the following sections.

As for the Groningen flight 0601, the prevalent wind is from southeast, and there is an angle between the designed flight transects and the prevalent wind direction. The flight track does not only cover Groningen city but also covers other urban areas. The west flight transects have three altitudes, but the other flight track on the north, south, and east has one altitude. A strong plume of N_2O and CH_4 appears on the southern side of west flight track and western side of south flight track, and a bit weak plume appears on the southern part of the east flight track. The southwest corner has the highest concentrations considering the whole flight track. Prior to the west and south flight transects, the vertical profile is performed once on the north flight track that shows lower concentrations compared to the whole flight track. The situations of the flight have three main issues, 1) the wind direction based on the vertical profile has a strong negative linear correlation with altitude, 2) the concentration of one side of the flight track did not decrease to the background level; as a result, the plume width derived from the flight track is actually smaller than the real plume width, and 3) the CH_4 concentration based on the vertical profile has a strong negative linear correlation with altitude.

As for the Utrecht flight 0917, the prevalent wind is from southwest, and the designed flight transects on southwest and northeast are almost perpendicular to the prevalent wind direction. The upwind and downwind transects at the same single altitude were both repeated twice exactly at the same locations. The plume of N_2O and CH_4 appeared on both upwind and downwind transects, and on the northwest track as well. In terms of time, upwind transects were performed, followed by downwind transects, and then one vertical profile. The situations of the flight have two main issues, 1) the PBL is still developing based on the vertical profile and 2) the CH_4 concentration during ascent within the PBL has a strong negative linear correlation with the altitude but does not during descent.

As for the Rotterdam flight 0906, the prevalent wind is from southwest (sea), and the designed flight track includes six parallel flight transects at a single altitude that are almost perpendicular to the prevalent wind direction. The distance between the first and last parallel transects is from the Rotterdam harbor to the east outside Rotterdam city, and following this direction, a clear concentration gradient of N_2O and CH_4 is observed. The flight transects extend to the sky over The Hague and Delft, since they are very close to Rotterdam city. A vertical profile is conducted after the six parallel transects and located southeast outside Rotterdam city, which is not far away from the intensively managed agricultural areas surrounding the Cabauw tower. The situations of the flight have three main issues, 1) the wind direction during descent has a strong linear correlation with altitude, 2) the concentration of both N_2O and CH_4 has a negative linear correlation with altitude based on the vertical profile, and 3) determination of plume width is difficult.

2.6.2. Flux calculation strategy

The downwind background is not appropriate to be used to determine the emissions of both N_2O and CH_4 from the urban areas for Groningen and Utrecht. When using the concentrations of the edges outside the downwind plume as background, the enhancements of N_2O and CH_4 relative to them are caused by the emissions from the areas within the footprints of downwind transects. Since strong plumes also appear on the upwind transects, they will have a large impact on the derived enhancements relative to downwind background, based on which the estimated emissions are from the areas beyond the targeted urban areas.

Selecting upwind transects as background is not appropriate to estimate N_2O emissions from the urban areas of Groningen and Utrecht. The upwind background was determined by averaging the concentrations of upwind transects within a range that was found by projecting the downwind plume following the reversed wind direction to

the upwind (south) transect. The urban N_2O emissions of Groningen and Utrecht are so small that the enhancements relative to upwind background cannot be detected due to the change of meteorological conditions. The developing PBL diluted the downwind N_2O concentrations for Utrecht, and the increasing perpendicular wind speed to the transects hindered the accumulation of the N_2O emissions for Groningen, leading to the undetectable N_2O enhancements for the two urban areas. We have found that the N_2O enhancements relative to upwind background are around zero or the background uncertainty. These meteorological conditions theoretically influence the atmospheric transport of CH_4 molecules, but CH_4 enhancements are detectable for the two urban areas since the CH_4 emissions are large enough to overcome the impact of meteorological conditions.

The emission estimates over the urban areas of Groningen and Utrecht are determined as the difference between the in-flow and out-flow fluxes. The in-flow and out-flow fluxes are derived separately for the upwind and downwind transects. For Groningen, both of the perpendicular wind speed and the plume width for the calculation of the "in-flow" and "out-flow" fluxes are different. The in-flow fluxes are derived from the south flight transect at one single altitude, while out-flow fluxes are averaged from the derived fluxes for the west flight transects at three altitudes. For Utrecht, the PBL height is different for "in-flow" and "out-flow" flux calculation, and both in-flow and out-flow fluxes are averaged from the fluxes that are derived individually for the two transects for each upwind and downwind direction.

The emissions from the Rotterdam urban area are estimated using upwind transect measurements as background. If the targeted area is strictly limited to the Rotterdam area, using downwind as background most likely leads to an underestimation of emissions. Using upwind background also has its limitation, and the appropriate selection of background is discussed in the following section.

2.6.3. The selection of background and its corresponding enhancements

As for the in-flow and out-flow fluxes in this study, the edges outside the plume on upwind and downwind transects are selected as background. Various methods such as the values averaged from artificially selected time range (Gvakharia et al., 2020), and interpolation between the outermost boundaries of flight legs (Kostinek et al., 2021) have been used to determine background in previous studies, and standard deviation has also been used as an indicator to separate the background from the plume (Fiehn et al., 2020). In this study, we used the standard deviation of the mean concentration within the spatial resolution as an indicator to separate the background from the plume. Based on the prevalent wind direction over Groningen, the west flight transects showing plume are chosen as downwind transects, and the south flight transect is chosen as the upwind transect.For Utrecht, the parallel flight transects that are almost perpendicular to prevalent wind direction is apparently recognized as the upwind and downwind transects.

As for Groningen, both upwind and downwind transects show relatively low and stable concentrations at a single edge, from which the mean concentration is selected as background, while the rest of the transect showing higher concentrations is recognized as plume. It is noticed that for the downwind transects at three altitudes, the boundary and background of the plume is determined separately for each altitude, thereby producing the enhancements for each downwind transect.

As for Utrecht, two edges outside the plume for each transect show concentration gradients, and a linear function established by the median concentration of each edge is used to determine background. The plume concentration subtracted by the modelled background value from the linear function is averaged to derive plume enhancements. As such, a unique enhancement is derived for each transect

As for Rotterdam, we number the six parallel transects from the first to the sixth based on the location from harbor to the city center, and the sixth flight transect is selected as the downwind transect. The upwind background is determined by projecting the plume of the downwind transect, following the reversed prevalent wind direction, to the second transect rather than the first transect, as the part of the first transect is over sea. In this transect the concentration is too low and cannot reflect the real inland background. It has to be noticed that the upwind

background could also have a bias relative to the real background, since the sampling was not in an ideal Lagrangian frame. Moreover, the downwind background determined by a linear function was not used for calculation in this study, because it was most likely affected by agricultural emissions. The concentration of the north edge does not have a significant difference of upwind background, but the south edge concentration does and higher than the north edge concentration. The higher concentration of the south edge is most likely enhanced by the agricultural emissions that come from the southeast areas outside Rotterdam city center. Those areas are mostly dominated by agriculture as the predominant emission source. The concentrations of the flight track near the center of the agricultural areas are also higher than those at urban areas.

2.6.4. Boundary layer depth and mixing

The PBL height is determined by the vertical profiles of the potential temperature, and when the potential temperature does not have a clear transition along the altitude, the PBL height has been determined by the vertical profile of trace gases. We note that it is not recommended to use the vertical profile of trace gas concentration to determine the PBL height in the "IG3IS Urban Greenhouse Gas Emission Observation and Monitoring Good Research Practice Guidelines". Besides the vertical profiles, the HYSPLIT model helps to evaluate if the PBL grows up or collapses.

The PBL depth remained constant for the flights over Groningen and Rotterdam, and the height in the equation is set as 1250 m and 1000 m respectively, while the PBL for the flight over Utrecht was developing. As for the Utrecht urban area, the vertical profile of potential temperature does not show a clear transition from the PBL to the troposphere, and the descent profile of trace gas concentrations shows a higher PBL than what the ascent profile shows, which indicate the PBL was developing throughout the flight. The simulated PBL height from HYSPLIT model also shows a trend of increasing height during the flight. We note that since the spatial resolution of the HYSPLIT model is not high enough for Utrecht urban areas, the model result is only used as a confirmation. The PBL height for the "in-flow" and "out-flow" flux calculation was determined by the ascent and descent profiles of trace gas concentrations being 650 m and 750 m, respectively. However, the "real" PBL height based on potential temperature for "in-flow" and "out-flow" flux calculation would be smaller than the height of 650 m that ascent profile shows because the vertical profile happened after upwind and downwind transects.

The ideal situation for applying the mass balance approach is that the emissions of N_2O and CH_4 have already mixed well within the PBL, which is often not the case in a real situation. The N_2O concentration within the PBL does not have a linear correlation, pointing to being well-mixed, with the altitude for Groningen and Utrecht, but does for Rotterdam, while CH_4 concentrations within the PBL are linearly correlated with the altitude for all three urban areas. The linear correlation only exists using the ascent profile for CH_4 for the flights over Groningen and Utrecht, while for the flight over Rotterdam, both ascent and descent profiles of N_2O and CH_4 show a linear correlation with altitude.

As for Rotterdam, the estimates presented here have already included that the plume is not well mixed. The vertical profile was conducted downwind, so we assumed that the observed downwind plume followed the same vertical distribution as the vertical profile and that the upwind background was vertically well mixed. A linear correlation between the mole fraction profile and altitude shown by the ascent profile was used to estimate downwind plume at multiple altitudes for both N_2O and CH_4. In addition, the enhancements for multiple altitudes were derived, based on which emissions were estimated. Furthermore, we also calculated the emissions assuming a vertically well-mixed downwind plume, which are ~4 % for N_2O and ~7 % for CH_4, larger than the estimates for not well-mixed plume. It has to be mentioned for the flight over Rotterdam that the concentrations of both N_2O and CH_4 decreased during descent. We speculate it could be caused by the heterogeneity of the source areas in combination with a changing wind direction to around 100° during descent.

For the cases of Groningen and Utrecht, we find that the vertical profiles cannot provide useful information to estimate the uncertainty caused by not well-mixed CH_4 mole fractions. As for Groningen, the vertical profile was conducted at background locations rather than near downwind plume locations and therefore cannot offer much

useful information about the vertical distribution of downwind CH_4 plume either. Besides this, out-flow fluxes have already considered the not well-mixed CH_4 mole fractions, as the downwind transects were measured at three different altitudes, and the out-flow flux of CH_4 is averaged from the fluxes derived from the transects at the three altitudes. As for Utrecht, quick dynamic vertical mixing and the lack of vertical profile below ~300 m hinder the use of vertical profiles to estimate the upwind and downwind plume. First, the PBL height determined by descent profile is larger than that determined by the ascent profile. Moreover, the simulated PBL height from the HYSPLIT model also validated that the PBL was developing during the course of flight, as shown below in Figure S18. The upwind transects were first flown, followed by downwind transects and then one vertical profile. During the period (11 ~ 12 UTC) when upwind and downwind transects were performed, the simulated PBL developed fast from ~600 m to ~1000 m, and when performing vertical profiles (12:30~13:00 UTC), the simulated PBL developed slowly from ~1200 m to ~1300 m. The dynamic vertical mixing is so quick that the vertical profile may not be suitable to infer the vertical distribution of upwind and downwind plume. Second, the vertical profile was not performed from ground to the height of ~300 m, and as a result, there was very limited information about the vertical distribution of CH4 below the shallow PBL with a height of ~650 m.

2.6.5. The wind direction and speed

The wind direction and speed are obtained from Skyarrow plane meteorological observations rather than modelled results considering the low accuracy of inverse modeling on a small spatial scale. One of the assumptions is that wind direction and speed should keep stable during the course of the flight. There is not a quantitative standard of the definition of 'stable wind', and based on the time series and vertical profiles of wind direction and wind speed, we can assume they are stable. The average wind speed and direction with standard deviation excluding vertical profiles are 7.1 $\pm$ 1.3 m/s and 275.2° $\pm$ 15.7 ° for Groningen, 3.9 $\pm$ 0.9 m/s and 69.3° $\pm$ 13.7° for Utrecht, and 3.7 $\pm$ 1.1 m/s and 70.4° $\pm$ 19.8° for Rotterdam. The average wind direction and wind speed is aimed for horizontal wind, and the degree of wind direction (North indicates 0° and clockwise South indicates 180°) indicates the direction that wind goes forward rather than originates from.

It is also required for the mass-balance approach that the wind should have a minimal vertical shear, but a quantified criteria to define a minimal shear was not found in previous studies. As for Groningen, the wind direction of the vertical profiles shows a strong linear correlation with altitude, and the average wind direction with the 1-σ standard deviation for the downwind transects at the high, middle, and low altitude is 292° ± 11°, 267° ± 8°, and 261° ± 9°. As for Utrecht, wind direction and speed were not found to be dependent on the altitude below the height at which the wind speed and direction have a sharp change. As for Rotterdam, the wind direction of the 1st transect has the smallest deviation compared to that of the other five transects; the average wind speed of 1st transect and 2nd transect is 5 m/s and 4.4 m/s, higher than the average wind speed 3.2 m/s of the other four transects.

In the flux calculation, we used the average wind direction and speed within the PBL for Utrecht and Rotterdam. For Groningen, the wind direction and speed have been used separately for the flux calculation for each transect.

2.6.6. The plume width

As for the estimated emissions from Groningen and Utrecht urban areas that are defined as the difference of "in-flow" and "out-flow" fluxes, the plume width is determined separately for the upwind and downwind transects. Since the flight track did not fully frame the plume from Groningen urban areas, the plume width is determined as the distance from the boundary between one edge and plume to the farthest point of the flight transects. As such, the determined plume width is smaller than the real width of the plume. Besides, the concentrations along the flight transects over Utrecht show the peaks with variable patterns, and the plume width is determined separately for the two upwind and downwind transects. As for Rotterdam, the plume width is determined from the 6th flight transect that is performed outside city center.

2.6.7 Uncertainty in the estimated emissions

The relative uncertainties in the mass balance flux estimates are derived by propagating the relative uncertainties from the several parameters for the flux calculation. Doing so, the comprehensive relative uncertainty of the flux estimates for Utrecht and Rotterdam sums the relative uncertainties of concentration enhancements, wind speed, wind direction, plume width, and PBL height in quadrature, while plume width uncertainty was not taken into account for the estimated emissions from Groningen urban areas. We already known that the flight track over Groningen did not frame the whole plume, and we are not certain about how much the real plume width is larger than the used value of plume width in the flux calculation.

The derived relative uncertainties of the estimated emissions in this study are the lower limits of the relative uncertainties since they do not take into account all the factors that might influence the emission values, such as the entrainment of free troposphere, the selection of background, and the assumption that the plume concentration is vertically mixed well within the PBL.

The uncertainties of the enhancements are derived by summing the uncertainties of the background and plume in quadrature. The background uncertainty is derived by summing the atmospheric variability and measurement precision in quadrature, and the atmospheric variability is represented in two ways depending on the background calculation. If the background is derived by the mean concentrations, the atmospheric variability is represented by the 1-σ standard deviation of the mean concentrations that are not apparently influenced by the emissions, while if the background is calculated from a linear function, the atmospheric variability is represented by the 1-σ standard deviation of the residuals between the modeled values from the linear function and the observed values. The plume uncertainty is represented by the measurement precision. Moreover, the wind speed and direction vary within a range in real conditions although we assume the wind is stable and used average wind speed and direction in the flux calculation. The wind speed uncertainty is represented by 1-σ standard deviation of the mean values, and the wind direction uncertainty is represented by 1-σ standard deviation of the cosine value of the angle between the wind direction and the direction perpendicular to flight transects. The uncertainties of plume width and PBL height are set manually as different constant values based on ‘eye-check’ for different flights.

The relative uncertainties of the estimated emissions from Groningen and Utrecht urban areas are derived by the propagation of the relative uncertainties of “in-flow” and “out-flow” fluxes, while for the Rotterdam urban areas, the relative uncertainties of the fluxes are derived separately for two situations were either upwind or downwind has been used as background. As for Groningen, the “out-flow” flux is the mean of the fluxes that are derived from the downwind transects at three altitudes, while for Utrecht, both of the “in-flow” and “out-flow” fluxes are the mean of the fluxes that are derived from the two upwind and two downwind transects. In these cases, the relative uncertainty of the “in-flow” or “out-flow” flux is the largest one of the relative uncertainties of the flux for each upwind/downwind transect.

As for Groningen, the relative uncertainties of enhancements, wind speed, and wind direction are derived separately for the downwind transects at the three altitudes and the upwind transect, while the relative uncertainty of the PBL height is the same because the PBL was stable during the course of the flight. The relative uncertainty of enhancements contributed the most to the relative comprehensive uncertainties of the “in-flow” and “out-flow” fluxes for N_2O; the relative uncertainty of wind direction contributed the most to the relative comprehensive uncertainty of the “in-flow” CH_4 flux, while the relative uncertainties of enhancements and wind speed contributed the most to that of the “out-flow” CH_4 fluxes. The relative uncertainties of PBL height contribute the least to the relative comprehensive uncertainties of the “in-flow” fluxes for both N_2O and CH_4, but the factors that contributed the least to the relative comprehensive uncertainty of “out-flow” fluxes are the PBL height or wind direction, depending on the flux derived from which downwind transect.

As for Utrecht, the relative uncertainties of wind speed and wind direction are the same for the “in-flow” and “out-flow” fluxes, while the other parameters’ uncertainties are different. The relative uncertainties of wind speed and enhancements are the top two largest among all parameters' relative uncertainties for both “in-flow” and “out-flow” fluxes of N_2O, and the wind speeds’ uncertainty contributed the most to the uncertainties of both “in-flow” and

"out-flow" fluxes of CH_4. The relative uncertainties of the "out-flow" fluxes that are derived using the two downwind transects data are the same for both N_2O and CH_4, while the relative uncertainties of the "in-flow" fluxes are different.

As for Rotterdam, only the enhancements' uncertainties are different for both N_2O and CH_4 fluxes using the upwind and downwind background, and the other factors' uncertainties are the same. The wind speed's relative uncertainty is the largest for the CH_4 fluxes using both upwind and downwind as background, and for the N_2O fluxes using the upwind as background, while for the N_2O flux using the downwind as background, the enhancements' relative uncertainty is the largest among all of the parameters' relative uncertainties.

3.2 Theoretical analysis

In the practice of performing a mass balance approach, the biggest challenge, especially for N_2O, is to gain a reliable enhancement. If the enhancement is larger than the uncertainty of background (in our case, the uncertainty is represented by the combination of background variability and measurement precision), we believe that the enhancement is reliable for mass balance estimation. Based on a simple theoretical calculation, we can estimate the minimum emissions that are required to gain reliable enhancements.

During theoretical analysis, we assume an ideal situation to perform mass balance estimation, in which the PBL is stable and mixed well, the wind direction and speed is constant, the plume for targeted areas is isolated with the plume for other areas, and designed flight transects are perpendicular to wind direction. If the emissions and background are constant, there are three main scenarios in which the enhancements are reduced, potentially being unreliable for mass balance calculation: 1) developing PBL dilutes the downwind concentrations, leading to reduced enhancements, 2) wider distributed plume means diluted concentrations, leading to reduced enhancements, and 3) larger wind speed carries more molecules of N_2O and CH_4 away from the sources and prevents them accumulating in an area, which caused low concentrations of downwind and smaller enhancements.

The background uncertainty that has taken into account both instrument precision and background variability was set as a threshold. The enhancements above that are considered to be reliable for mass balance estimation. We define the smallest detectable emission as the flux commensurate with an enhancement equal to the background uncertainty, assuming plume and meteorological conditions as in Table S10. Because the meteorological conditions are variable during flight, the calculated detectable minimum emissions are shown in a range separately for three urban areas.

Table S1. The dimensions and key parameters of the new big-volume airborne active AirCore.

Length	285 m
Tubing	Stainless steel
Outer diameter (OD)	4.762 mm (3/16 in.)
Wall thickness	0.177 mm (0.007 in.)
Coating	SilcoNert 1000, by Restek Inc.
AirCore tubing weight	5.7 kg
The AirCore volume in 1 atm	4.3 L
Power consumption	~5 W

Table S2. Overview of the flights over Groningen, Utrecht, and Rotterdam.

Flight.no	Date	AirCore sampling [UTC]	Sampling rate [sccm]	Areas	Onboard LI-7810	Analysers for AirCore measurements	PBL depth[a] [m]	Average wind[b] direction [deg]	Average wind[b] speed [m s^{-1}]
1	2020-08-20	11:26 – 12:26	37	Groningen	NO	Two CRDS in series	500	/	/
2	2020-09-02	08:56 – 11:21	37	Utrecht	working	Two CRDS in series	Growing up	/	/
3	2020-09-07	12:44 – 14:53	34	Groningen	working	Two CRDS in series	500-300, collapse	/	/
4	2020-09-14	09:29 – 12:09	30	Utrecht	working	Two CRDS in series	Not really developed yet	/	/
5	2020-09-15	09:33– 11:32	40	Utrecht	working	Two CRDS in series	Not really developed yet	/	/
6	2021-05-31	13:34 – 15:52	40	Groningen	NO	Two CRDS in series	1100	275±18	6.5±1.2
7	2021-06-01	13:40 – 15:40	40	Groningen	NO	Two CRDS in series	1250	273±16	7.2±1.3
8	2021-09-17	10:55 – 13:14	40	Utrecht	working	QCL	Grow up from 650 m to 750 m	69±14	3.9±0.9
9	2021-09-28	10:50 – 13:09	40	Utrecht	working	QCL	Grow up from 650 m to 1000 m	15±10	6.6±1.1
10	2022-08-30	11:49-14:22	37	Rotterdam	working	QCL	1250	234±8	8.8 ± 1.0
11	2022-09-01	12:19-14:59	32	Rotterdam	working	QCL	1600	261±13	6.3±1.2
12	2022-09-05	11:16-13:47	37	Rotterdam	working	QCL	1450	360±9	4.9±1.3

13	2022-09-06	12:22–14:54	37	Rotterdam	working	QCL	1000	70±20	3.7±1.1

[a]The PBL depth is derived simply by eye-check of the vertical profile.

[b]The average wind direction and speed is averaged from the wind direction and speed during the whole flight, excluding the wind above the PBL depth. And the wind direction indicates where wind will come toward.

Table S3. The diffusion volume with a unit of mL at STP, ΔV_{diff}, for multiple gas species with a typical storage time of 4 hours

N_2O	CH_4	CO_2	CO
20	23	20	22

Table S4. The smearing volume with a unit of mL at STP for the measurements performed by G5310, G2401-m, and QCLS for each flight

Flight	analysers	Measurement frequency	Flow rate (sccm)	Equivalent volume for each measurement at STP	Equivalent cell volume at STP	ΔV_{smear}
2020-09-02	G5310	0.5 Hz	56	1.87	5.5	5.5
	G2401-m	0.25 Hz		3.73	1.6	3.73
2020-09-07	G5310	0.5 Hz	47.5	1.58	5.5	5.5
	G2401-m	0.25 Hz		3.17	1.6	3.17
2020-09-14	G5310	0.5 Hz	54.8	1.83	5.5	5.5
	G2401-m	0.25 Hz		3.65	1.6	3.65
2020-09-15	G5310	0.5 Hz	48.8	1.63	5.5	5.5
	G2401-m	0.25 Hz		3.25	1.6	3.25
2021-05-31	G5310	0.5 Hz	66.7	2.22	5.5	5.5
	G2401-m	0.25 Hz		4.45	1.6	4.45
2021-06-01	G5310	0.5 Hz	66.7	2.22	5.5	5.5
	G2401-m	0.25 Hz		4.45	1.6	4.45
2021-09-17	QCLS	1 Hz	62	1.03	7.2	7.2
2021-09-28	QCLS	1 Hz	56	0.93	7.2	7.2
2022-08-30	QCLS	1 Hz	54	0.9	9.1	9.1
2022-09-01	QCLS	1 Hz	45	0.75	9.1	9.1
2022-09-05	QCLS	1 Hz	45	0.75	9.1	9.1
2022-09-06	QCLS	1 Hz	46	0.77	9.1	9.1

Table S5. The spatial resolution [km] of the measured concentrations for multiple species during all of the flights

	N_2O	CH_4	CO_2	CO-G5310
2020-09-02	1.1	1.3	1.1	1.3

2020-09-07	1.5	1.8	1.5	1.7
2020-09-14	1.5	1.7	1.5	1.7
2020-09-15	1.1	1.3	1.1	1.3
2021-05-31	1.3	1.5	1.3	1.4
2021-06-01	1.4	1.6	1.4	1.5
2021-09-17	1.2	1.4	1.2	1.4
2021-09-28	1.2	1.4	1.2	1.3
2022-08-30	1.4	1.6	1.4	1.5
2022-09-01	1.4	1.6	1.4	1.6
2022-09-05	1.4	1.5	1.3	1.5
2022-09-06	1.5	1.7	1.5	1.6
Spatial resolution [km]	(1.1, 1.5)	(1.3, 1.8)	(1.1, 1.5)	(1.3, 1.7)

Table S6. The CH_4 comparison of Li-7810 measurements and the AirCore measurements

	Before optimal		After optimal		Change percent (%)	
Flight	Correlation coefficient	RMSE	Correlation coefficient	RMSE	Correlation coefficient	RMSE
2020-09-02	0.6403	33.1101	0.998	2.5272	55	92
2020-09-07	0.9944	4.9019	0.9991	2.5094	0.5	49
2020-09-14	0.9983	5.2748	0.9993	3.565	0.10	32
2020-09-15	0.9863	34.317	0.9999	4.3202	1.4	87
2021-09-17	0.9985	3.2686	0.9989	2.8551	0.04	13
2021-09-28	0.994	5.7831	0.9955	5.5662	0.15	3.8
2022-08-30	0.916	5.9857	0.9772	4.3718	6.7	27
2022-09-01	0.9926	3.0979	0.9929	3.0951	0.03	0.1
2022-09-05	0.9672	5.0631	0.9913	2.8624	2.5	43
2022-09-06	0.9334	10.7216	0.9985	1.8521	7	83

Table S7. The in-flow and out-flow fluxes (kg hr^{-1}) with uncertainties

	Groningen		Utrecht	
	In-flow flux	Out-flow flux	In-flow flux	Out-flow flux
N_2O	427.7±332.6	522.7±285.1	158.4±63.4	190.1±63.4
CH_4	6336±3974	8870±2765	2419±806	3859±1094

Table S8. The average enhancements (ppb) relative to the mean concentration of upwind transects

Urban areas	N_2O background*	N_2O enhancement	CH_4 background *	CH_4 enhancement
Groningen	337.1±0.2	0.1±0.2	2027±1	11±2
Utrecht	338.5±0.1	0	2123±2	16±2
Rotterdam	336.9±0.2	0.9±0.2	2019±2	17±2

*The background uncertainty is the combination of atmospheric variability and measurement precision. Atmospheric variability is 1-σ standard deviation of the mean upwind concentrations that are not apparently influenced by the emissions from upwind sources.

Table S9. The relative uncertainty of each parameter and the estimated fluxes

Urban areas	Flux for each transect	enhancements		Wind speed	Wind direction	Plume width		PBL height	Comprehensive relative uncertainty of the fluxes	
		N_2O	CH_4			N_2O	CH_4		N_2O	CH_4
Groningen	In-flow	50%	12.5%	11.8%	26%	/	/	4%	58%	31%
	Out-flow at high altitude	50%	12.5%	12.3%	9.7%	/	/	4%	53%	20%
	Out-flow at middle altitude	40%	11.1%	9.2%	1.7%	/	/	4%	41%	15%
	Out-flow at low altitude	40%	11.1%	16.7%	2%	/	/	4%	44%	21%
Utrecht	In-flow.1	16.7%	4.3%	23.1%	10.6%	11.1%	10%	7.7%	33%	29%
	In-flow.2	25%	15%	23.1%	10.6%	11.1%	16.7%	7.7%	38%	35%
	Out-flow.1	20%	3.6%	23.1%	10.6%	7%	7%	6.7%	34%	27%
	Out-flow.2	20%	3.6%	23.1%	10.6%	7%	7%	6.7%	34%	27%
Rotterdam	Downwind as bg	33.3%	16.7%	29.7%	13%	14.3%	14.3%	10%	50%	40%
	Upwind as bg	11.1%	11.8%	29.7%	13%	14.3%	14.3%	10%	38%	39%

Table S10. The range of input values for each variable in theoretical mass balance calculation

	N_2O background uncertainty (smallest reliably detectable enhancement) [ppb]	CH_4 background uncertainty (smallest reliably detectable enhancement) [ppb]	PBL height [m]	Plume width [km]**	WS [m s^{-1}]***	Pressure [hpa]****	Temperature [kelvin]****
Groningen	0.1	1	1250	18.7~20.6	5.0~9.0	914.56	289.56
Utrecht	0.1	1~3*	650~750	18.5~23.1	2.3~5.4	965.01	290.64
Rotterdam	0.2	2	1000	14.6	2.0~5.6	967.94	293.83

* The uncertainty of CH_4 background is 1 ppb and 3 ppb for out-flow and in-flow flux calculation.
** Under the condition that flight track is perpendicular to wind direction.
*** The limits of the range of wind speed are the 5% and 95% quantiles of wind during horizontal flights.
**** The average atmospheric pressure and temperature under the PBL height. Note that the pressure shown in the table includes water vapor pressure, but average dry air pressure was used for mass balance calculation due to measured mole fractions of dry sample.

Table S11. The estimates (kg hr^{-1}) of the emissions of N_2O and CH_4 from urban areas.

		Groningen	Utrecht	Rotterdam
N_2O	Total Emissions	95.0±90.3	31.7±16.5	364.3±142.6
	Emissions per capita	$4x10^{-4}$	$8.8x10^{-5}$	$5.6x10^{-4}$
	Detectable minimum emissions*	63.4~142.6	15.8~63.4	31.7~95.0
CH_4	Total Emissions	2534±1774	1440±628	2419±922
	Emissions per capita	0.011	0.0040	0.0037
	Detectable minimum emissions*	253~507	63~645	132~363

* The range of detectable minimum emissions reflects the varying meteorological conditions encountered during the flights.

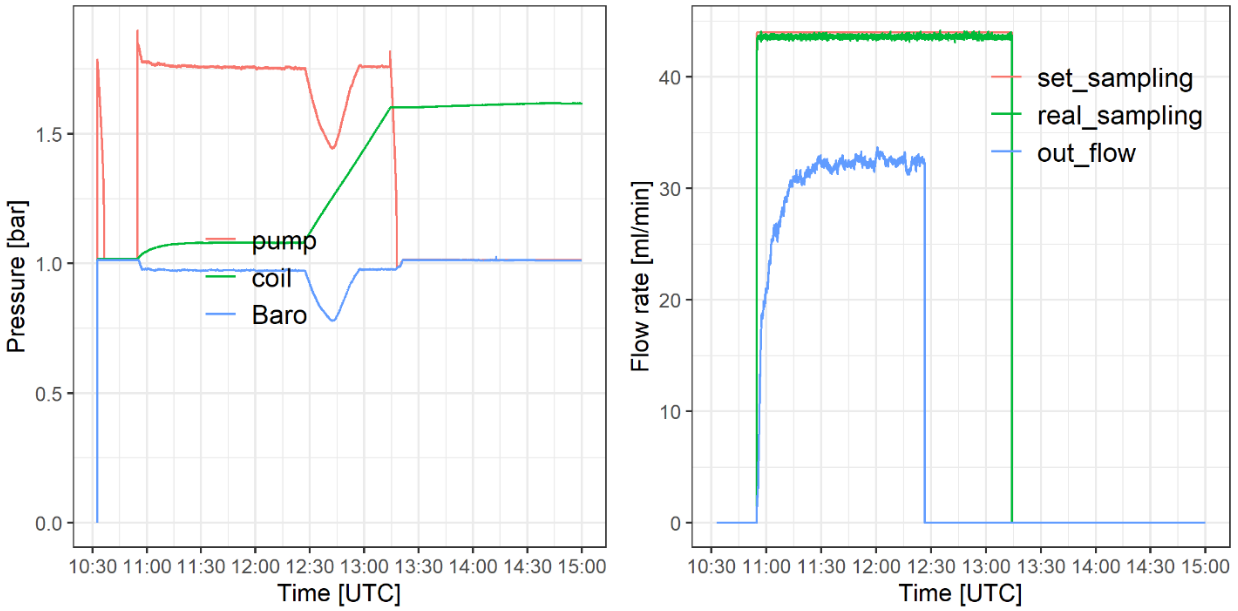

Figure S1. Take an example of the flight on September 17th to show the parameters change of the AirCore. (a) The change of coil pressure and ambient pressure during the flight (b) The sampling rate and out flow rate.

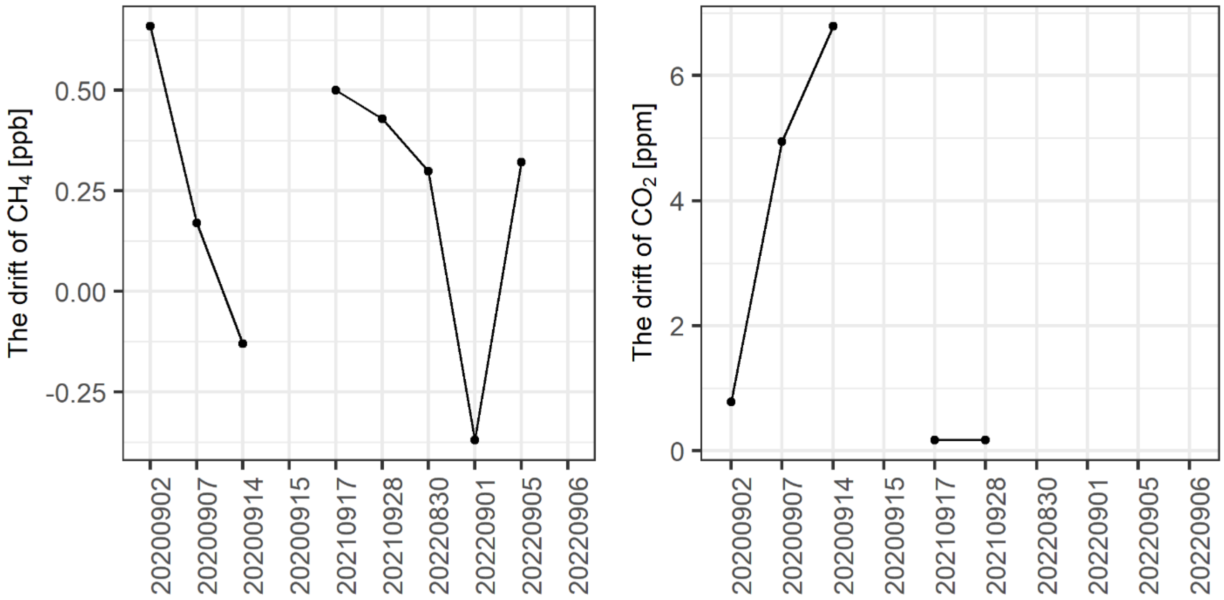

Figure S2. The drift of LI-7810 measurements before and after a flight for CH_4 and CO_2.

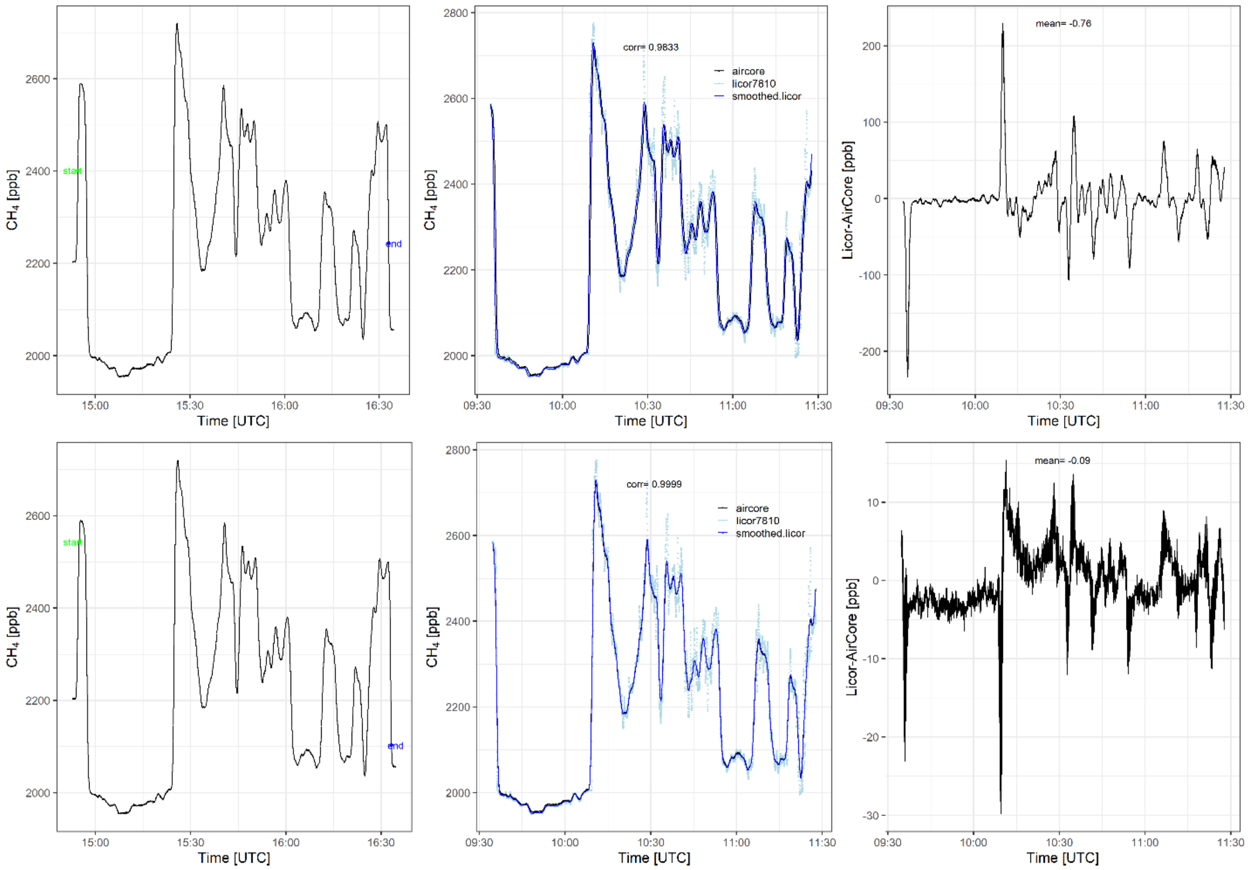

Figure S3. The comparison of the new airborne active AirCore by in-situ CH_4 measurements on September 15, 2020 before (top panel) and after (bottom panel) optimizing the AirCore retrieval.

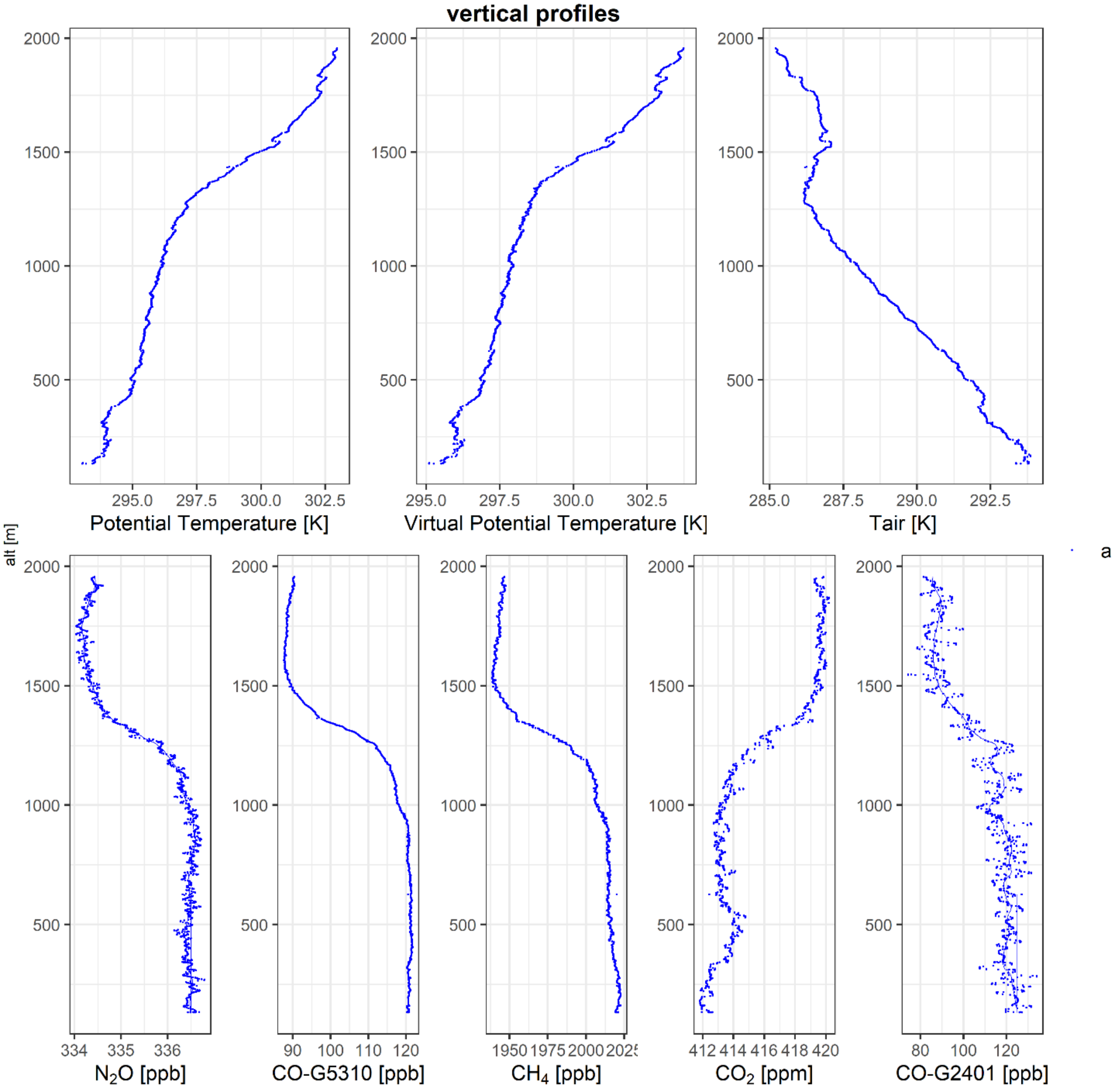

Figure S4. We only had the vertical profiles during spiraling up for the flight 0601. The mole fractions were measured by two Picarro analysers in series with two CO measurements and without COS measurements.

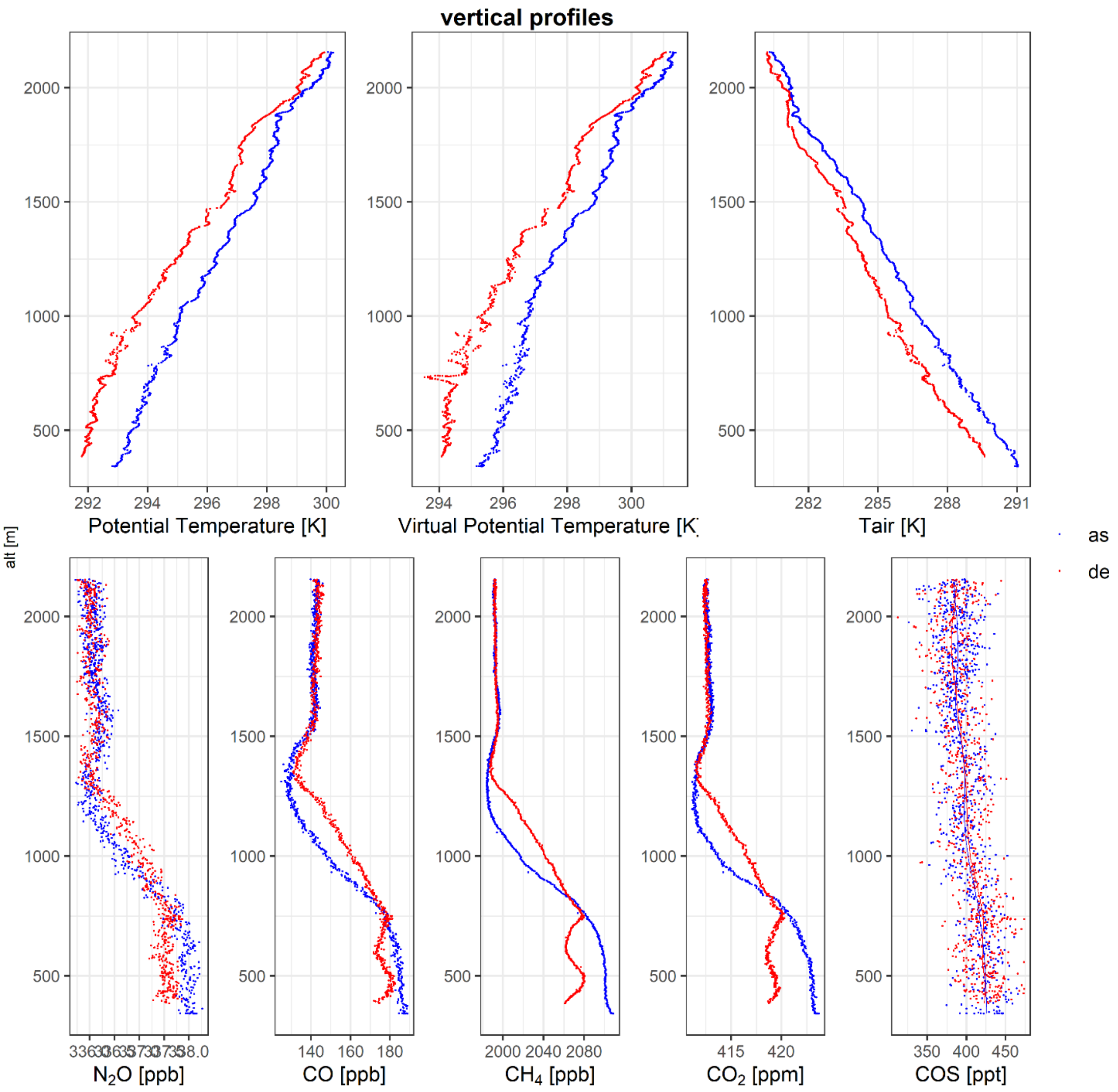

Figure S5. The verticals profiles for the flight 0917. Blue indicates the ascent and red indicates the descent. The mole fractions were measured by a QCLS with COS measurements.

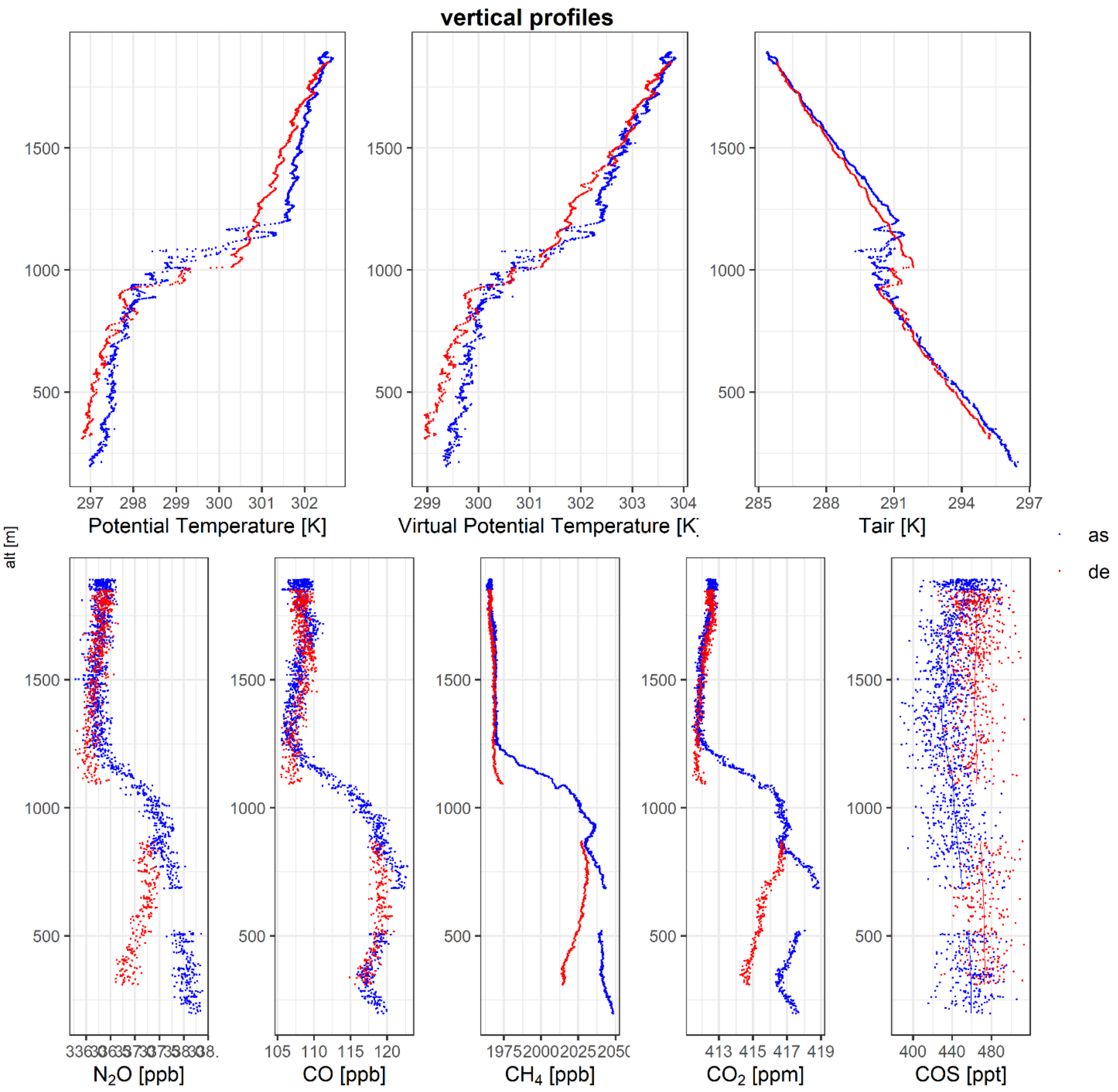

Figure S6. The vertical profiles for the flight 0906. Blue indicates the ascent and red indicates the descent. The mole fractions were measured by a QCLS with COS measurements.

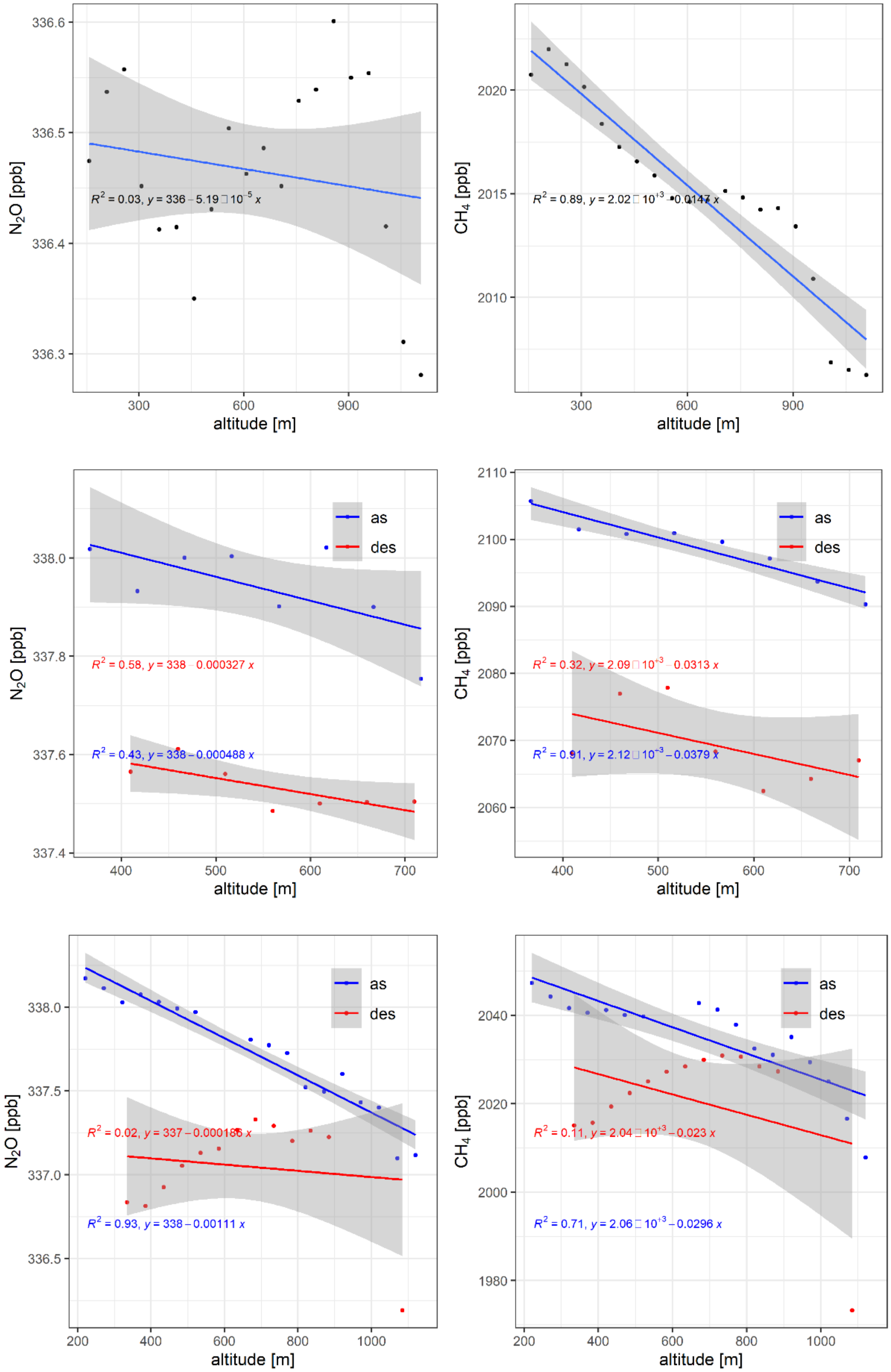

Figure S7. The correlation between the mole fractions of N_2O and CH_4 within the PBL and the altitude for the flight 0601 (top), 0917 (middle), and 0906 (bottom). The mole fractions are averaged with an interval of 50 meters.

Groningen flight: 0601

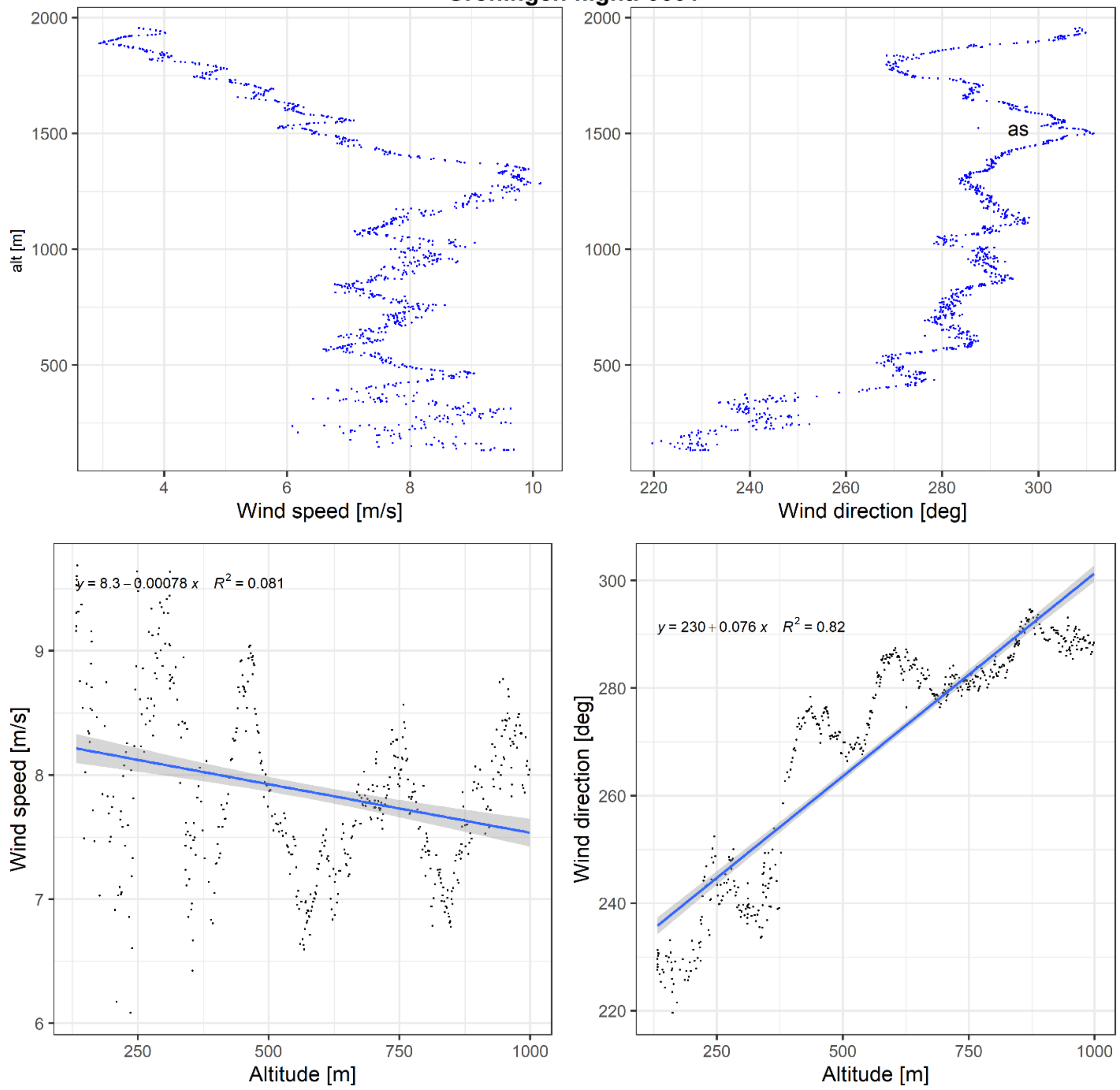

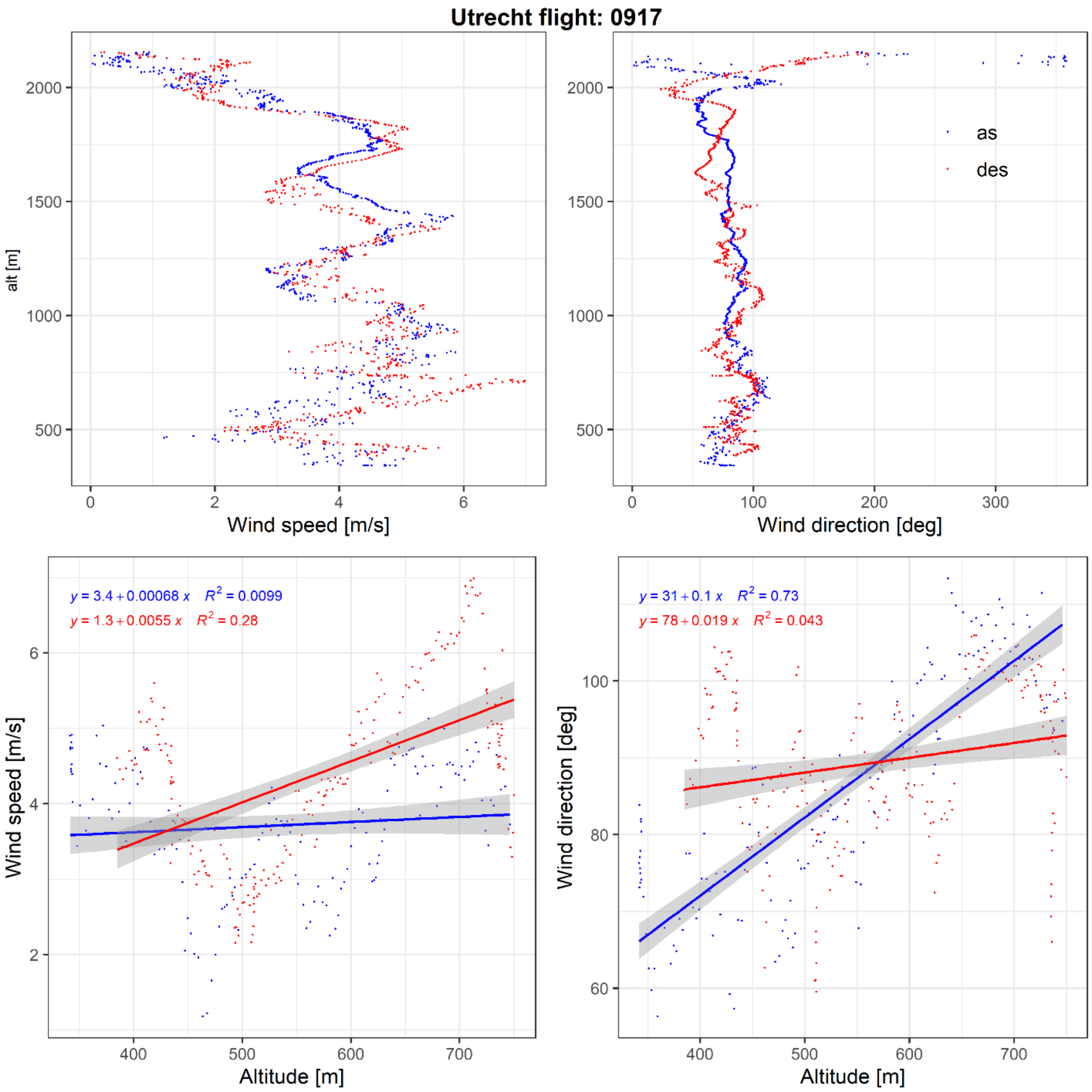
Utrecht flight: 0917
alt [m]
Wind speed [m/s]
Wind direction [deg]
as
des
y = 3.4 + 0.00068 x R² = 0.0099
y = 1.3 + 0.0055 x R² = 0.28
y = 31 + 0.1 x R² = 0.73
y = 78 + 0.019 x R² = 0.043
Wind speed [m/s]
Altitude [m]
Wind direction [deg]
Altitude [m]

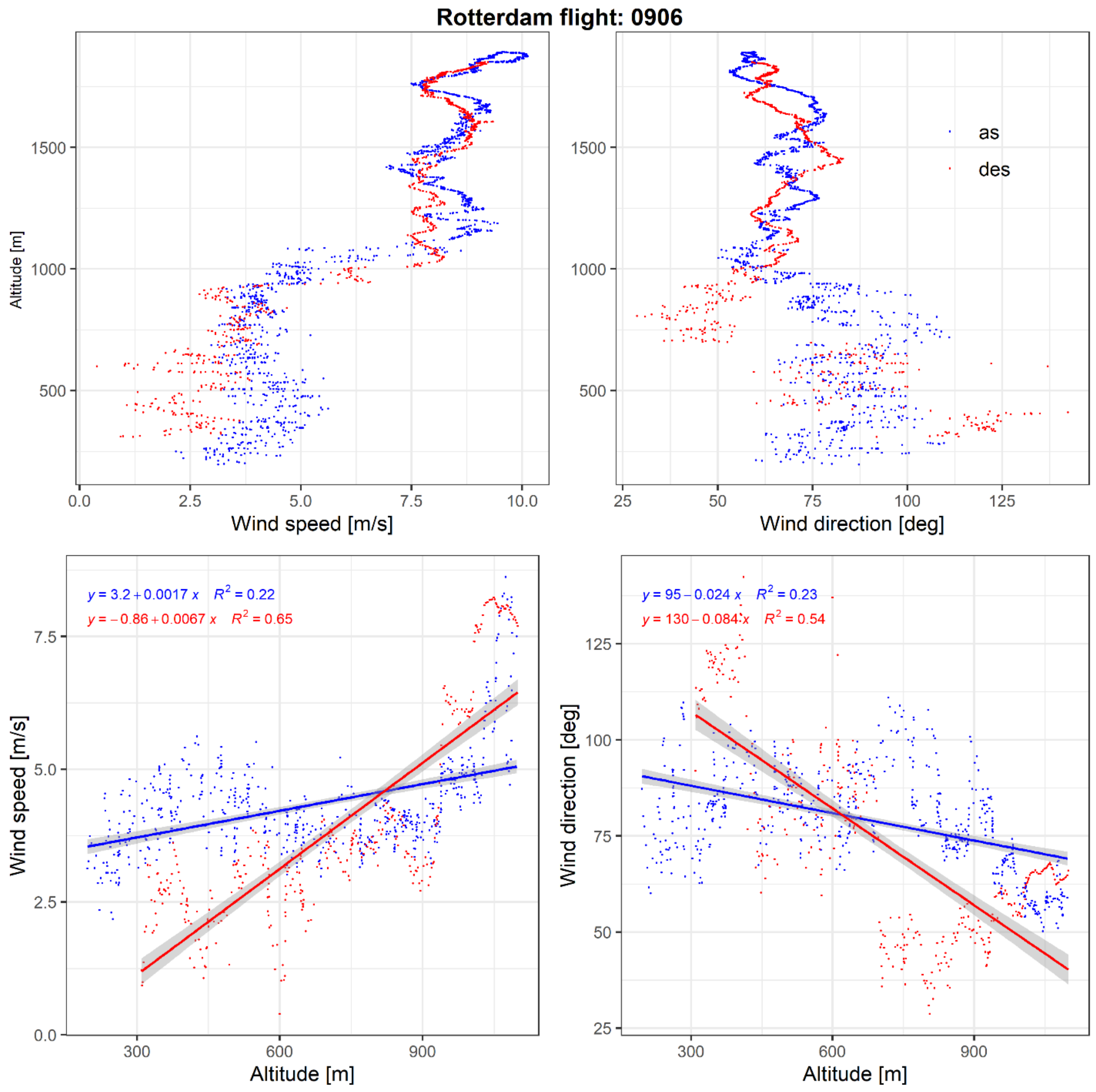

Figure S8. The correlation between wind speed and direction within the PBL and the altitude for the flight 0601, 0917, and 0906.

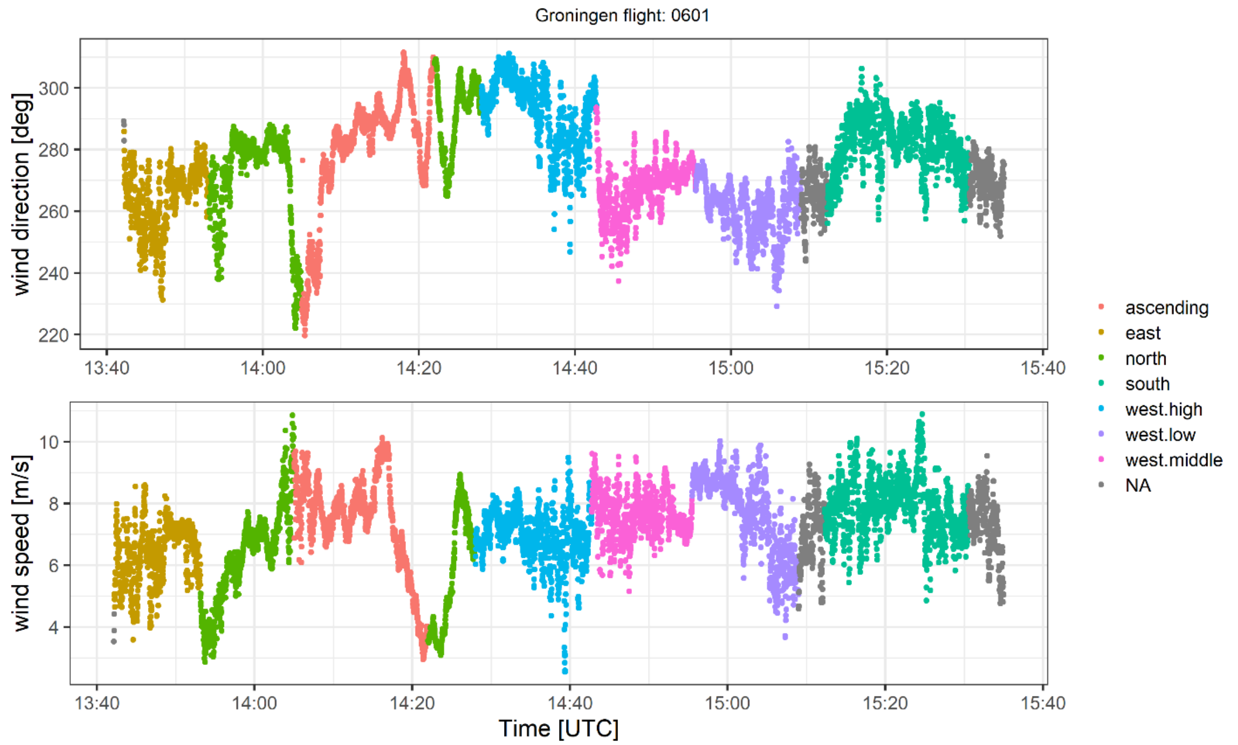

Groningen flight: 0601
wind direction [deg]
wind speed [m/s]
Time [UTC]
13:40
14:00
14:20
14:40
15:00
15:20
15:40
ascending
east
north
south
west.high
west.low
west.middle
NA

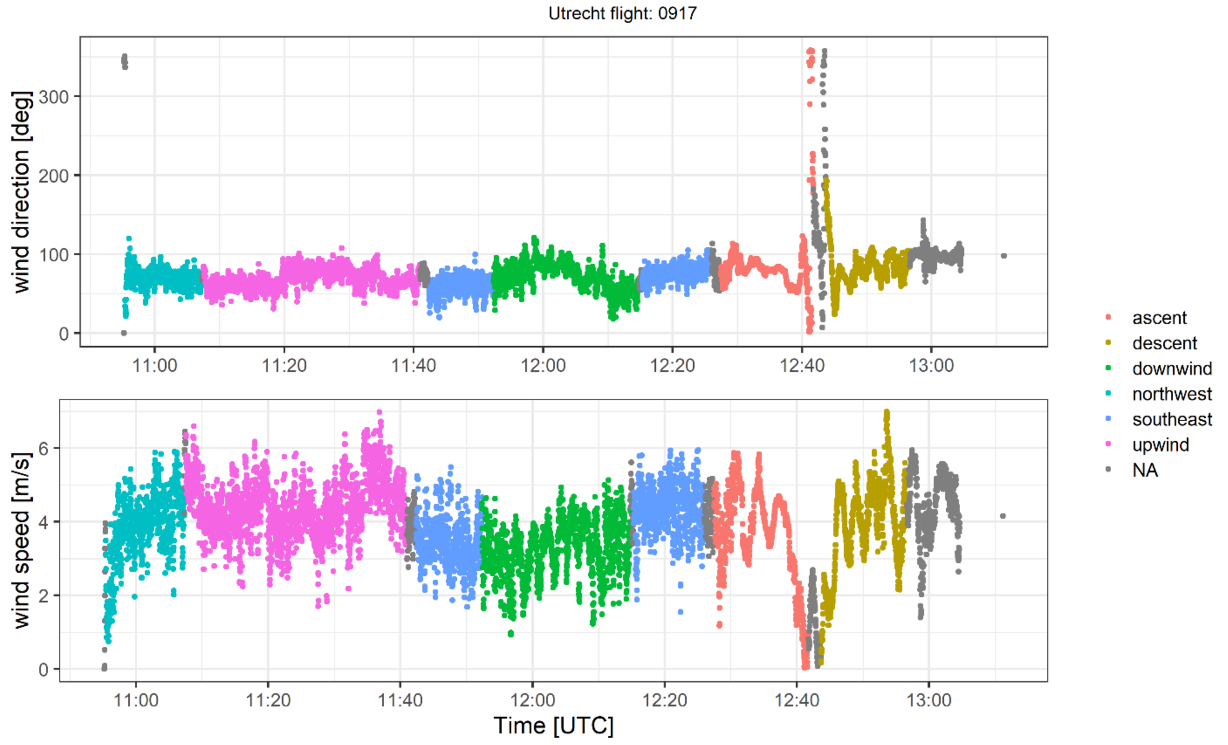

Utrecht flight: 0917
wind direction [deg]
wind speed [m/s]
Time [UTC]
11:00
11:20
11:40
12:00
12:20
12:40
13:00
ascent
descent
downwind
northwest
southeast
upwind
NA

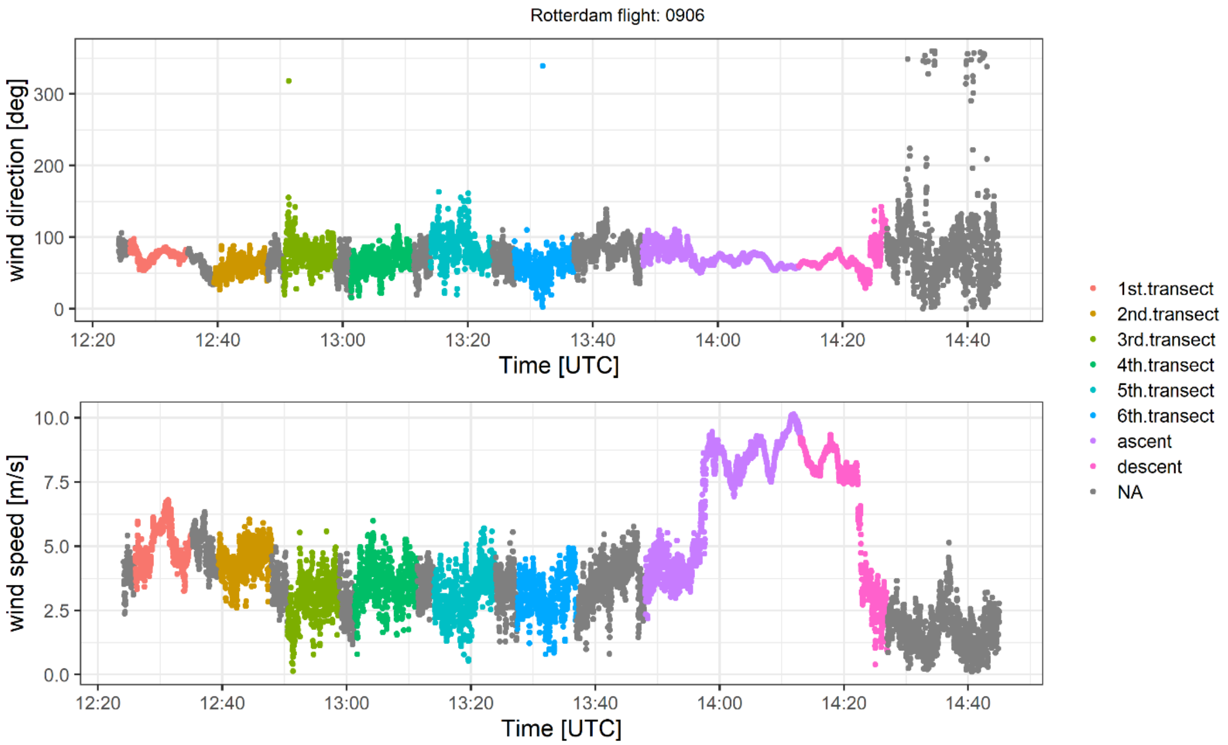

Figure S9. The time series of wind speed and direction for the flight 0601, 0917, and 0906.

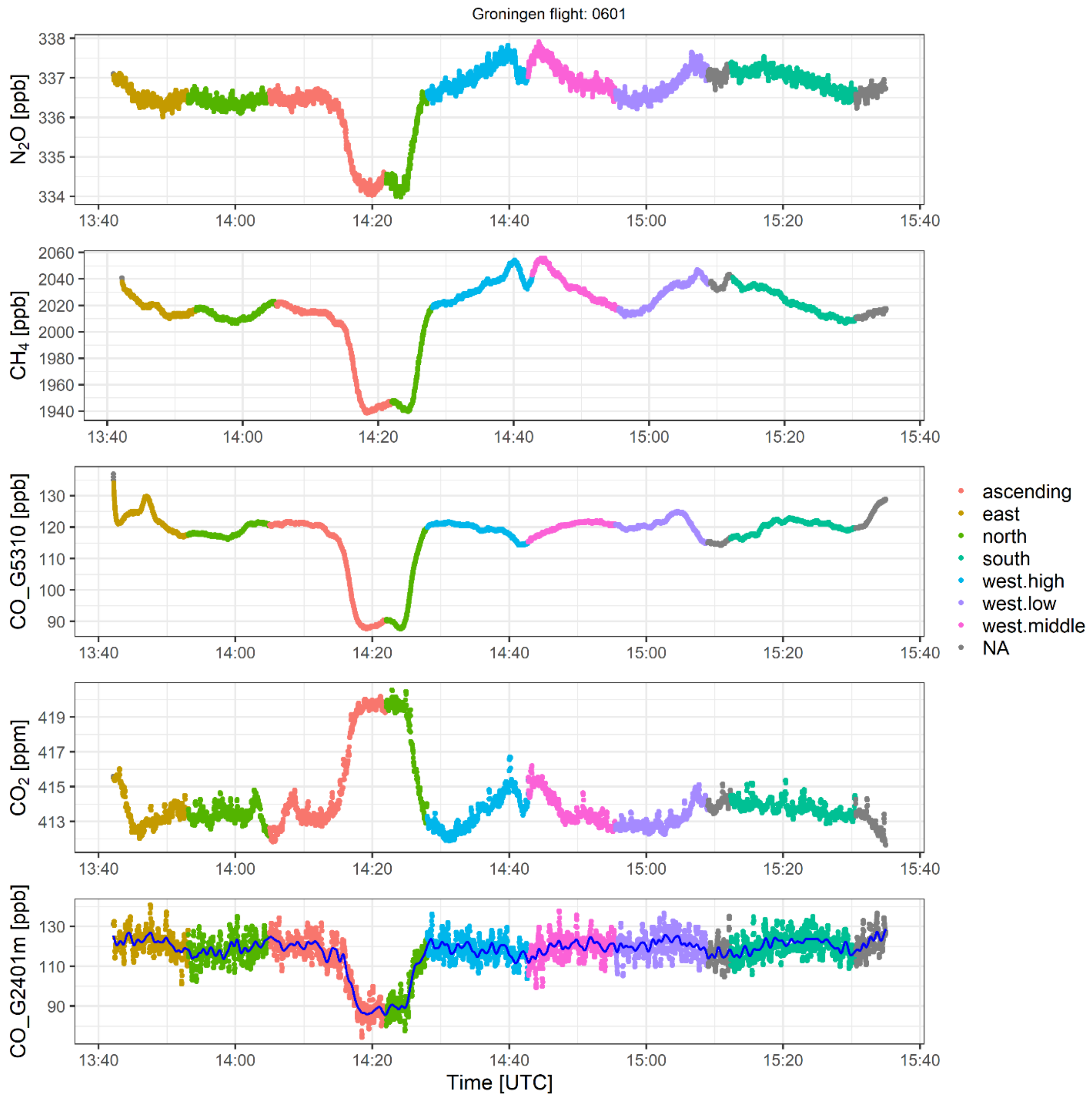

Figure S10. The time series of trace gas species for the flight 0601.

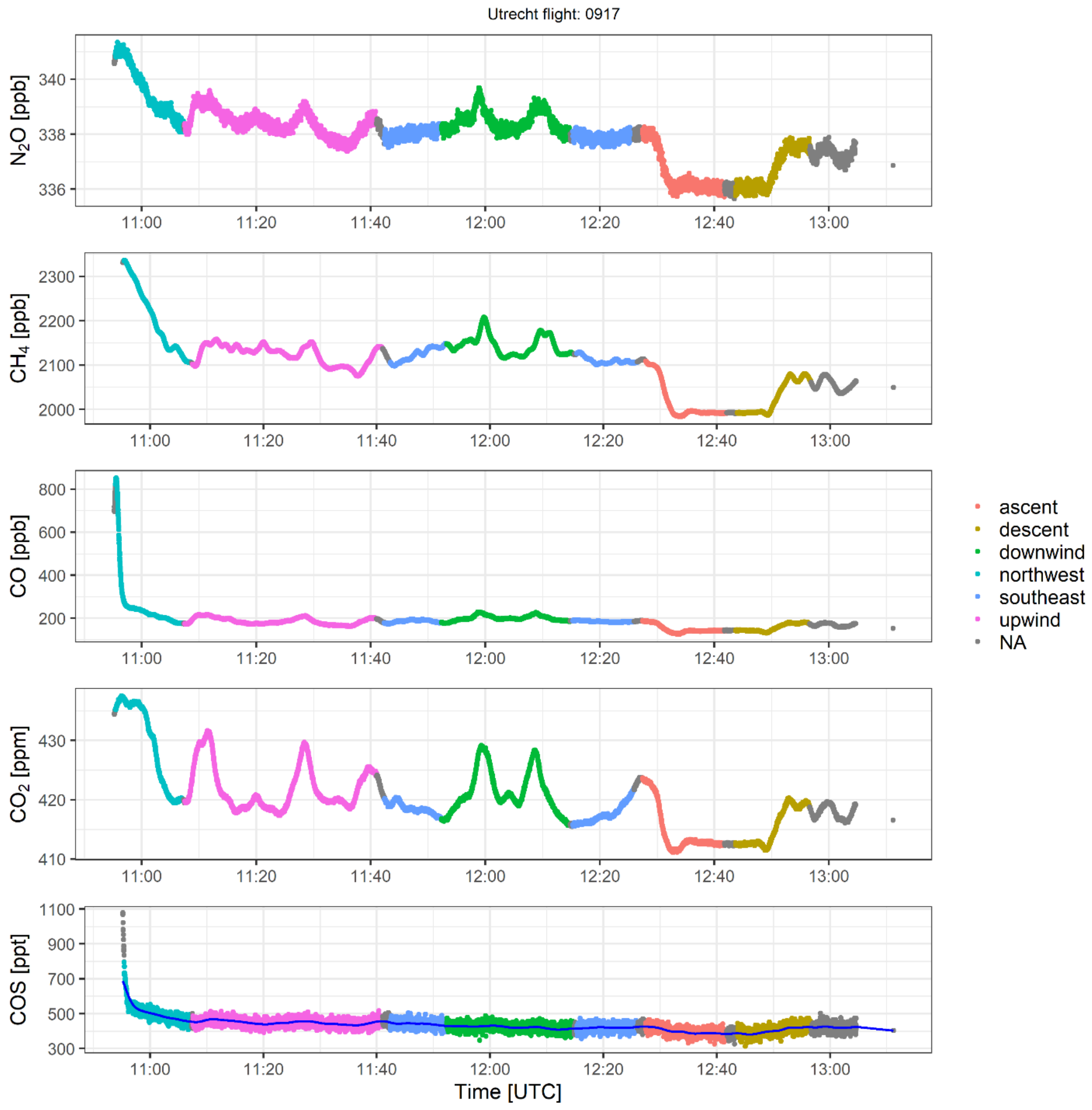

Figure S11. The time series of trace gas species for the flight 0917.

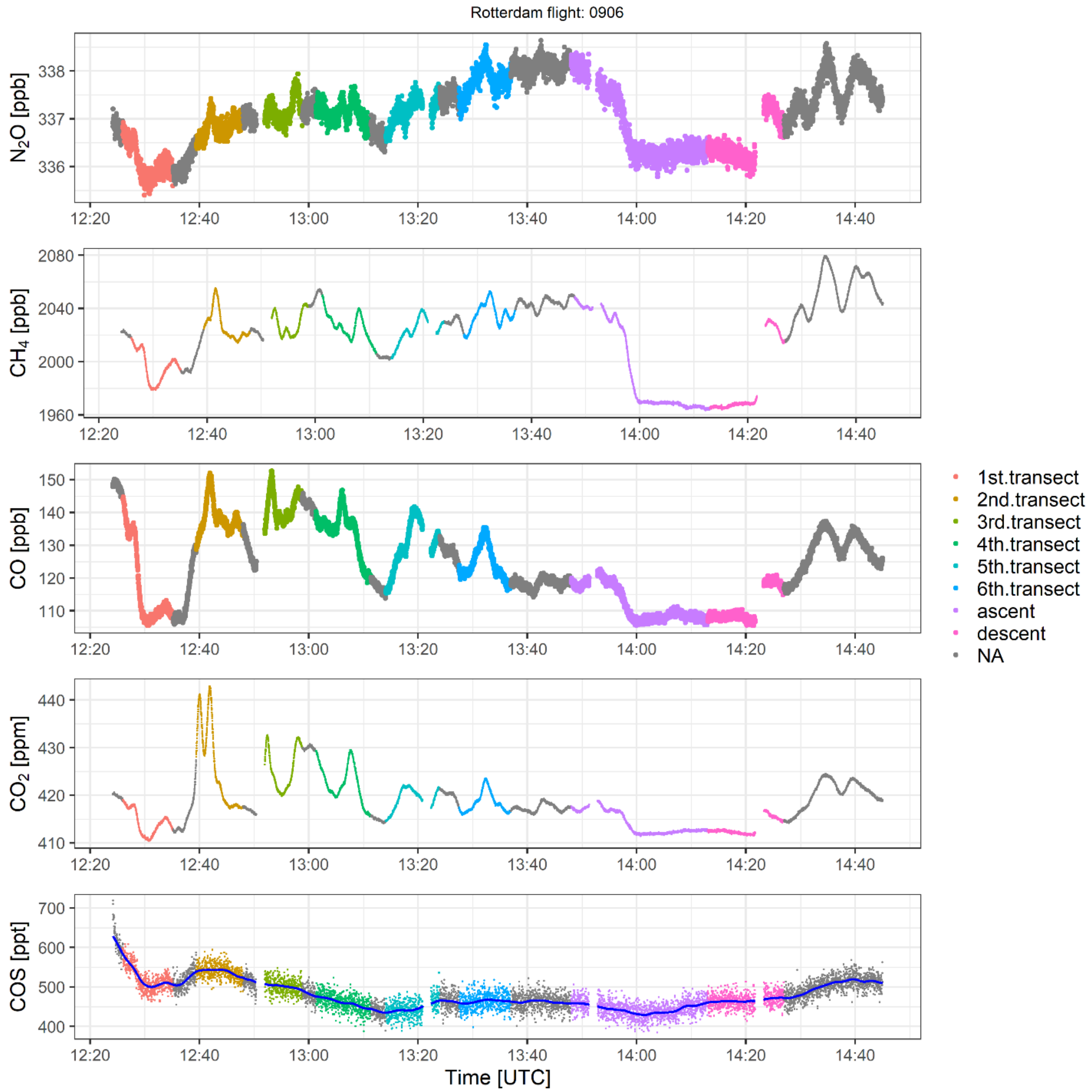

Figure S12. The time series of trace gas species for the flight 0906.

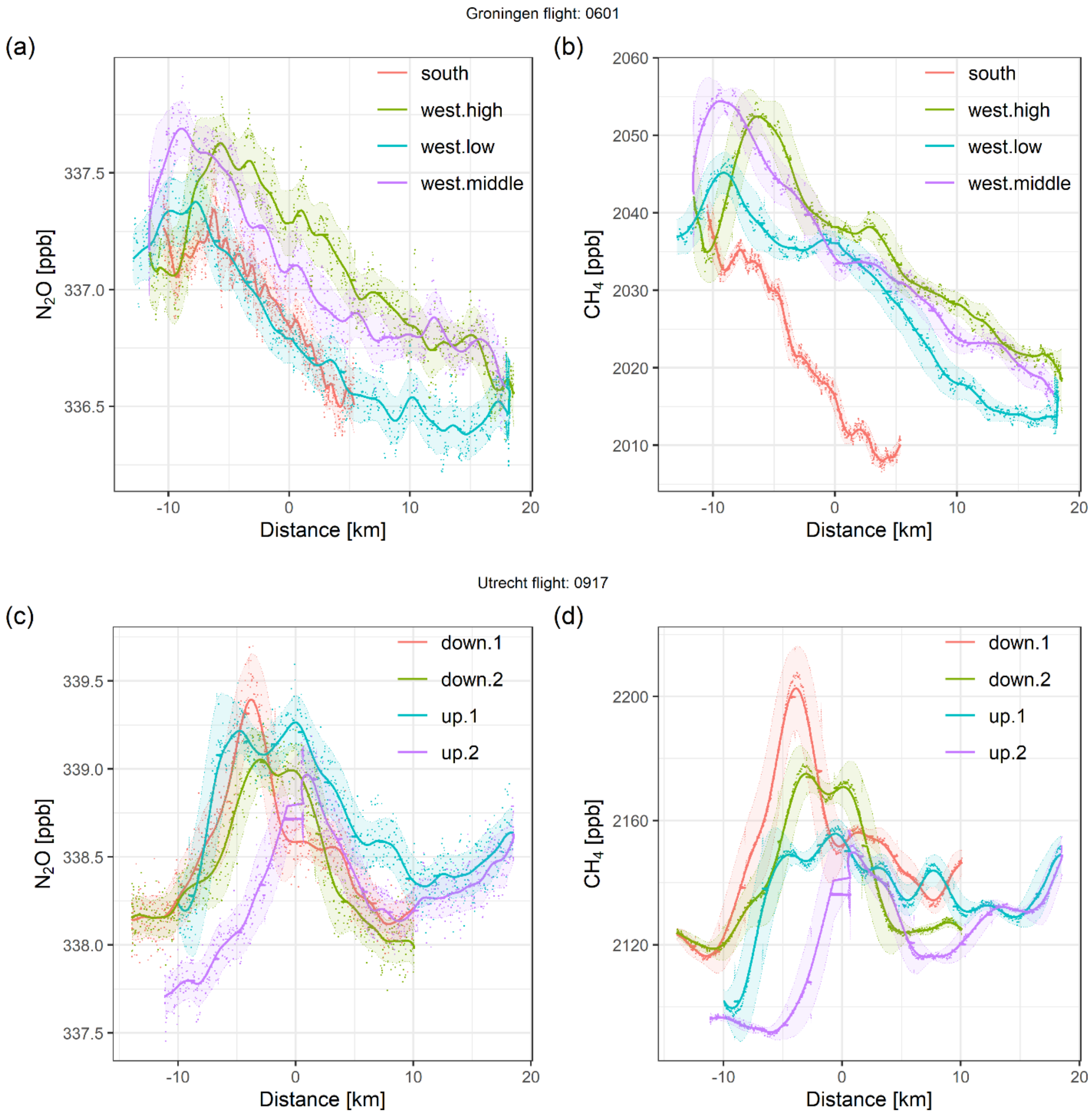
Groningen flight: 0601
(a)
(b)
south
west.high
west.low
west.middle
N2O [ppb]
CH4 [ppb]
Distance [km]
Utrecht flight: 0917
(c)
(d)
down.1
down.2
up.1
up.2
N2O [ppb]
CH4 [ppb]
Distance [km]

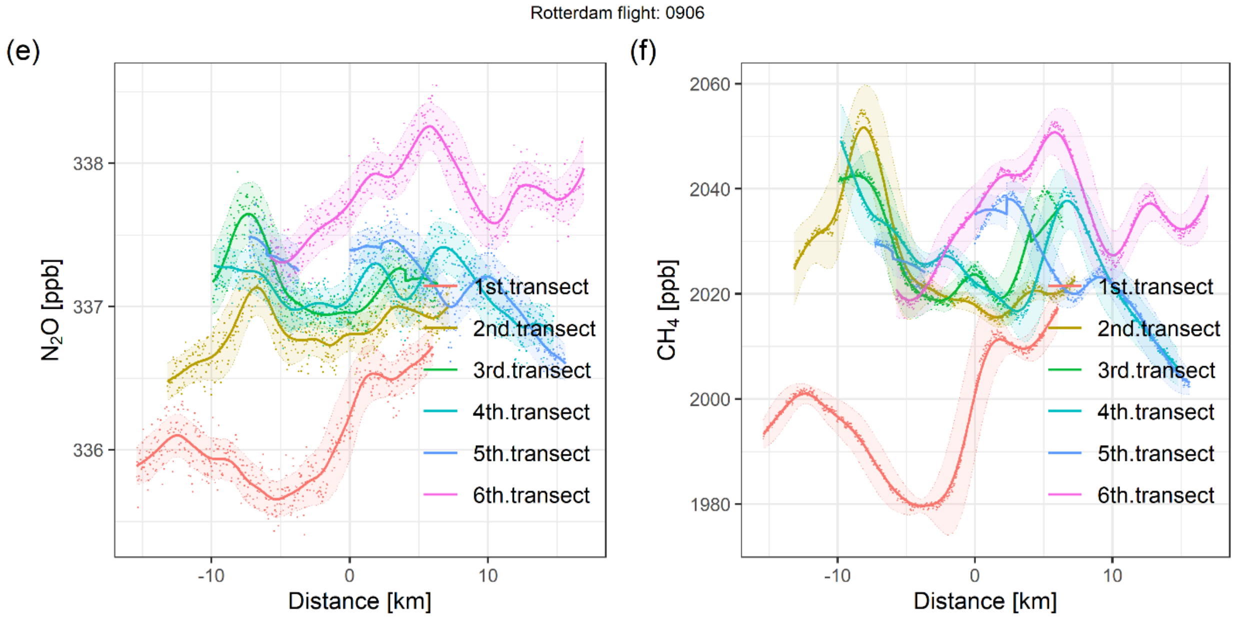

Figure S13. The mole fraction of N_2O and CH_4 along the transects perpendicular to wind direction for Groningen (top), Utrecht (middle), and Rotterdam (bottom). The distance indicates how far the points relative to arbitrary zero points along a virtual plane perpendicular to wind direction. The arbitrary zero points on each transects are the cross points following wind direction. The distance from negative values to positive values indicates latitude is larger for Groningen, while for the cases of Utrecht and Rotterdam, it is the other way around. In the Groningen flight, the west flight track was recognized as downwind transect and the concentration at high, middle, and low altitude was shown in green, purple, and blue colours, respectively; the south flight track was recognized as upwind transect, and the mole fraction was shown in red colour. In the Utrecht flight, the concentration of two repeated upwind transects at exactly same locations and altitude are shown in blue and purple, and in the same way, the concentration of two downwind transects are shown in red and green. In the Rotterdam flight, the six horizontal transects at the single altitude are numbered from one to six following the wind direction and shown in red, earth yellow, green, cyan, blue, and pink, respectively.

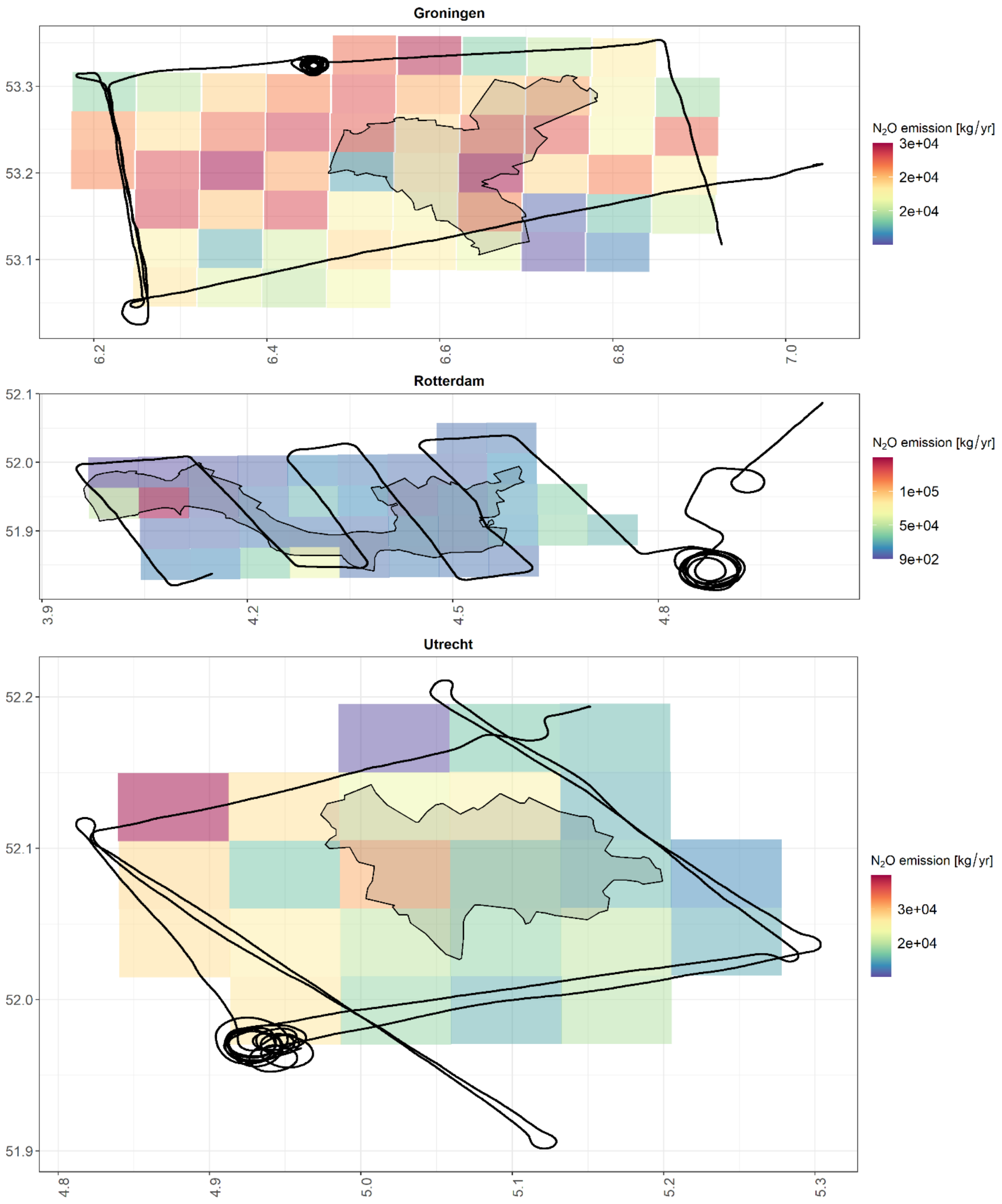

Figure S14. The Dutch N_2O emission inventory grid map of Groningen, Utrecht, and Rotterdam. The colour bar from blue to red indicates the emissions from low to high, the black line indicates the flight track, and the shaded area indicates the municipality boundary.

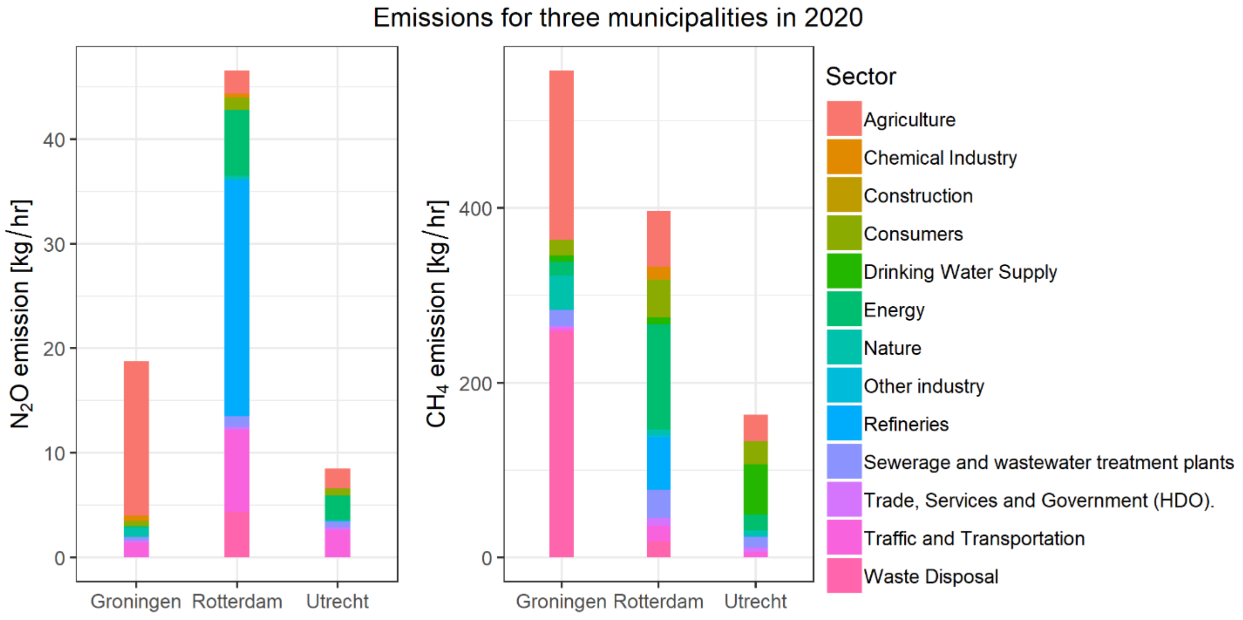

Figure S15. The inventory comparison of three municipalities of Groningen, Utrecht, and Rotterdam for the year 2020. The height of the bars indicate the total emissions of N_2O and CH_4 registered for the three municipalities, which are the sum of the emissions from variable sources that are shown by different colours.

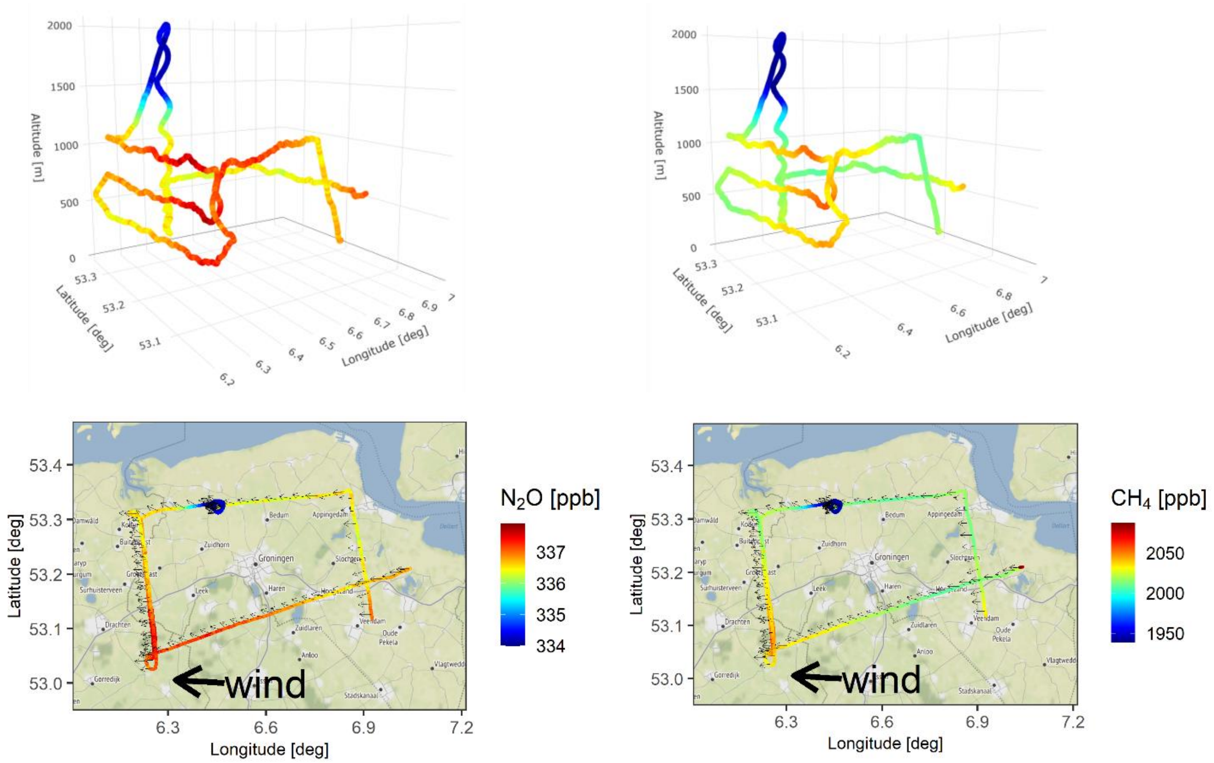

Figure S16. The 3D flight track and 2D concentration map of retrieved AirCore measurements for N_2O (left column) and CH_4 (right column) for the flight 0601. The mean wind direction during the course of flight below the PBL

height is shown by a bold arrow and the one-minute averaged wind direction is indicated by arrows along the flight tracks. The west transects were flown at ~1100 m, ~750 m, and ~450 m.

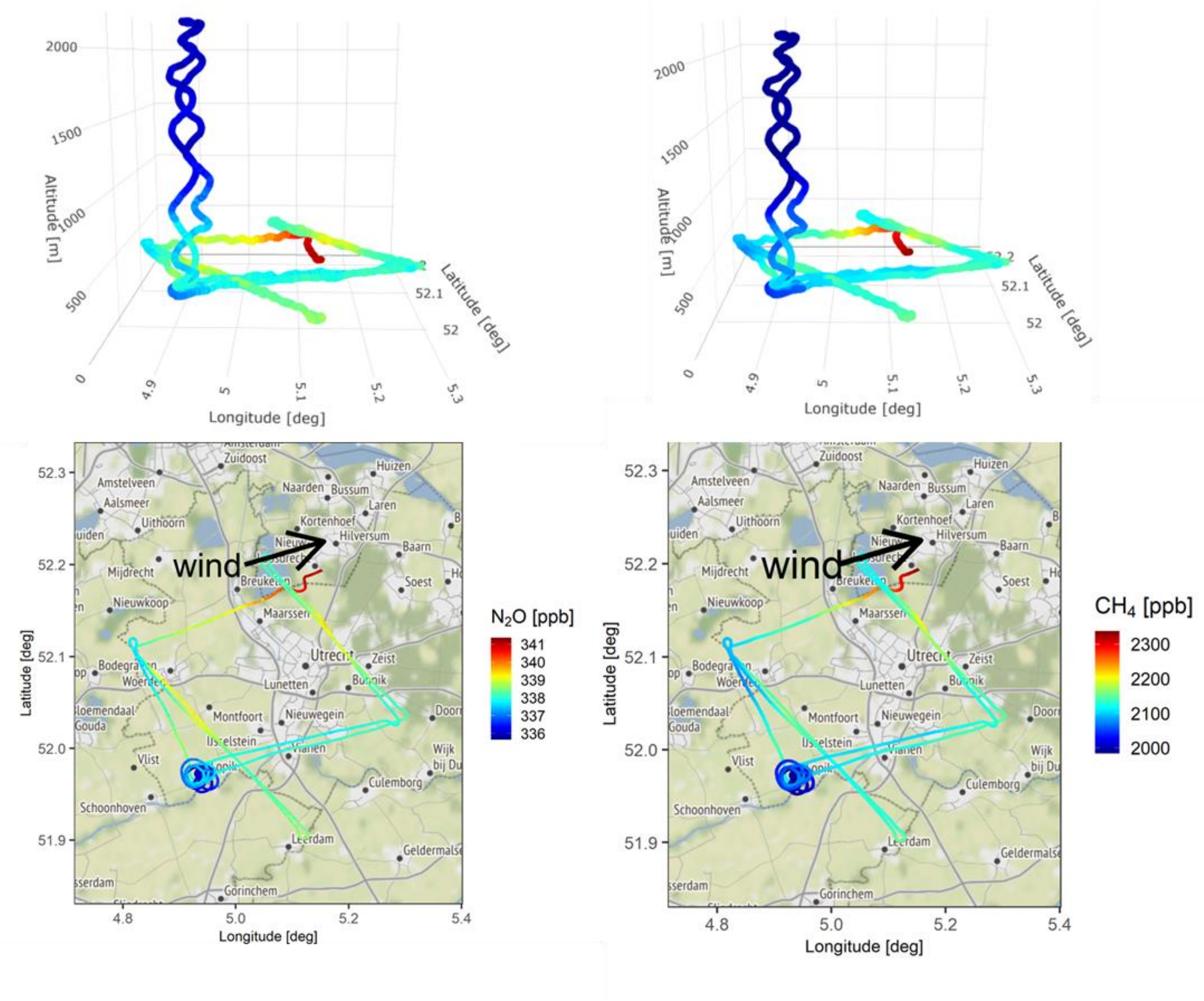

Figure S17. The 3D flight track and 2D concentration map of retrieved AirCore measurements for N_2O (left column) and CH_4 (right column) for the flight 0917.

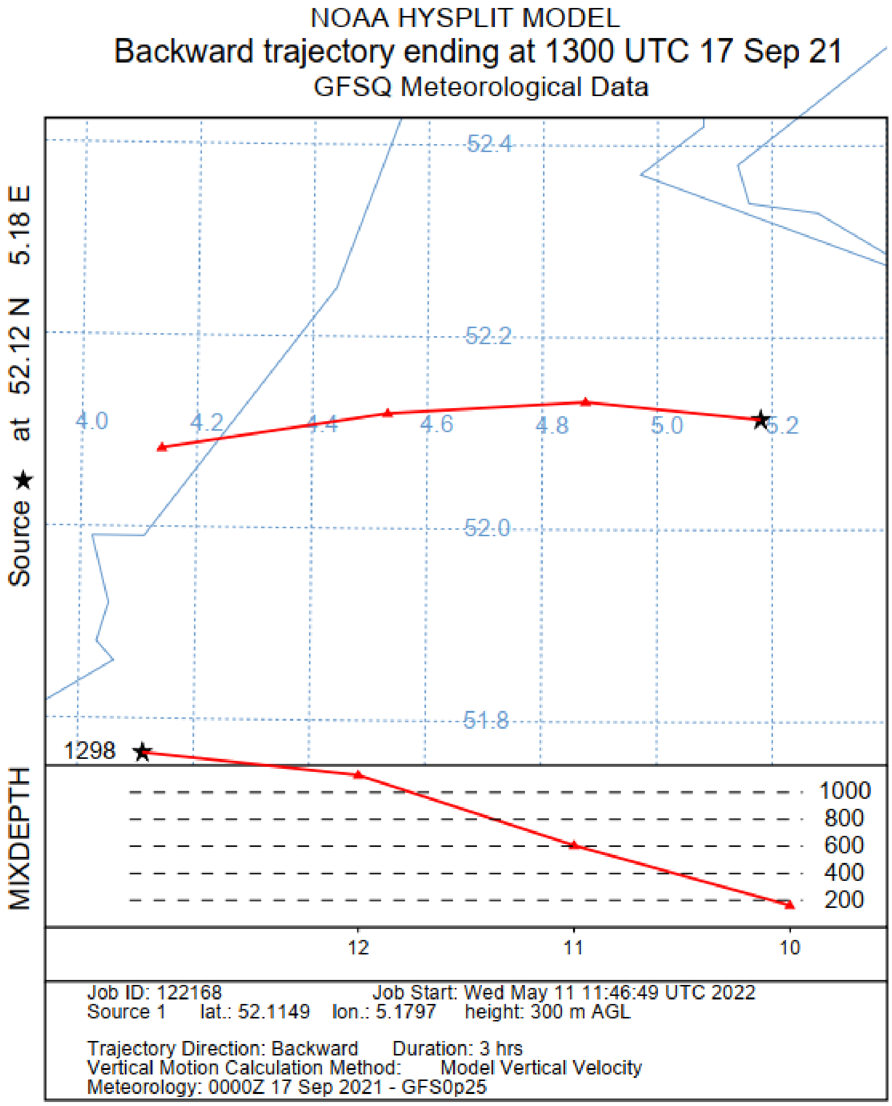

Figure S18. The PBL height simulated by NOAA HYSPLIT model for 17 September 2021.
